# Supplementary material for: Interventions promoting occupational balance in adults: A systematic literature review
Source: PLoS One. 2025 Jun 5;20(6):e0325061. doi: 10.1371/journal.pone.0325061 (PMC12176295; doi:10.1371/journal.pone.0325061)
Supplement: S2 Table — (DOCX) [file pone.0325061.s002.docx]

**Supporting information**

**S2 table. Amendments to protocol**

| **Protocol Section** | **Rationale** |
| --- | --- |
| Title | The focus on mental health turned out to be too narrow after conducting our preliminary search. |
| Review question | We accordingly also broadened the research questions to the adult population in general. |
| Searches | The search string as well the search strategy were adapted based on the decision to explore adults instead of adults with mental illnesses in this review. A more precise timeframe of the search was added (studies from 2000 to 2024). |
| Types of study to be included | We shifted from our original plan to conduct a mixed-method review to conducting a purely quantitative systematic review, as our initial search revealed few qualitative studies and great heterogeneity in quantitative publications. To improve the quality of the review, we decided to exclude qualitative and mixed-methods studies. |
| Participants | We changed the information provided to the population of adults instead of adults with mental illnesses accordingly. |
| Intervention | We further specified the inclusion criteria and eliminated the term “time-use“. |
| Risk of Bias | The quality assessment section was adjusted due to the fact that only quantitative studies will be involved. |
