# Supplementary material for: Interventions promoting occupational balance in adults: A systematic literature review
Source: PLoS One. 2025 Jun 5;20(6):e0325061. doi: 10.1371/journal.pone.0325061 (PMC12176295; doi:10.1371/journal.pone.0325061)
Supplement: S3 Table — (DOCX) [file pone.0325061.s003.docx]

**Supporting information**

**S3 Table. Search string**

| **PubMed** |
| --- |
| (("patient*" OR "client*" OR "participant*" OR "adult*" OR "person*" OR "women" OR "men" OR "grown-up*" OR "individual*" OR caregiv*”) |
| AND |
| ("therap*" OR "intervention*" OR "teach*" OR "educat*" OR "method*" OR "train*" OR “promot*” OR "program*" OR “occupational therapy”) |
| AND |
| ("occupational balanc*"OR “balance of occupation*)) |
| **CINAHL** |
| (("patient*" OR (MH "Patients+") OR "person*" OR "participant*" OR "client*" OR "individual*" OR "women" OR "men" OR "woman" OR (MH "Women+") OR "man" OR (MH "Men+") OR "grown-up" OR "adult" OR (MH "Adult+" OR “caregiv*”) |
| AND |
| ("therap*" OR “occupational therap*” OR "treatment*" OR "intervention” OR "counseling" OR "counselling" OR "method*" OR "teach*" OR "train*" OR "educat*" OR "promot*" OR "program*" OR "health education" OR “rehabilitation”) |
| AND |
| (“balance of occupation*” OR “occupational balance”)) |
| **The Cochrane Library** |
| (([Women] OR [Men] OR [Patients] OR patient* OR client* OR participant* OR adult* PR person* OR women* OR men* OR grown-up* OR individual* OR caregiv*) |
| AND |
| ([Occupational Therapy] OR [Therapeutics] OR [Health Educators] OR (therap* OR intervention* OR teach* OR educat* OR method* OR train* OR promot* OR program* OR (occupational NEXT therapy)) |
| AND |
| (occupational NEXT balanc*)) |
| **Embase** |
| ((“adult” OR “patient” OR "participant") |
| AND |
| ("therapy" OR "intervention" OR "education" OR "occupational therapy") |
| AND |
| ("occupational balance")) |
