# Supplementary material for: Interventions promoting occupational balance in adults: A systematic literature review
Source: PLoS One. 2025 Jun 5;20(6):e0325061. doi: 10.1371/journal.pone.0325061 (PMC12176295; doi:10.1371/journal.pone.0325061)
Supplement: S4 Table — (DOCX) [file pone.0325061.s004.docx]

| **Supporting information**  **S4 Table. All studies identified in the literature search** | | | |
| --- | --- | --- | --- |
| **No.** | **Ref Vancouver** | **Tags** | **Reason for exclusion** |
| 1 | Aas MH, Austad VM, Lindstad MØ, Bonsaksen T. Occupational Balance and Quality of Life in Nursing Home Residents. Physical & Occupational Therapy in Geriatrics. 2020;38(3):302-14. | Excluded after Title and Abstract Screening |  |
| 2 | Ahlstrand I, Larsson I, Larsson M, Ekman A, Hedén L, Laakso K, et al. Health-promoting factors among students in higher education within health care and social work: a cross-sectional analysis of baseline data in a multicentre longitudinal study. BMC Public Health. 2022;22(1):1-15. | Excluded after Title and Abstract Screening |  |
| 3 | Akyurek G, Abaoglu H, Colak DF, Bumin G. The effects of occupational therapy intervention on homeless adults living in homecare house. Journal of the Neurological Sciences. 2019;405:86. | Excluded after Fulltext Review | Article not peer-reviewed |
| 4 | Anaby DR, Backman CL, Jarus T. Measuring occupational balance: A theoretical exploration of two approaches. Canadian Journal of Occupational Therapy. 2010;77(5):280-8. | Excluded after Title and Abstract Screening |  |
| 5 | Andresen EM, Cannell MB, Akhtar WZ, Barney KF. CHAPTER 3 - Looming disease burden associated with the aging process: Implications for occupational therapy. In: Barney KF, Emerita C, Perkinson MA, editors. Occupational Therapy with Aging Adults: Mosby; 2016. p. 28-40. | Excluded after Title and Abstract Screening |  |
| 6 | Arcand-Dusseault C, Egan M. Occupations and personal projects: A comparison of the concepts. British Journal of Occupational Therapy. 2015;78(5):303-10. | Excluded after Title and Abstract Screening |  |
| 7 | Arcand-Dusseault C, Egan M. Personal Projects Analysis in occupational therapy research: A scoping review. British Journal of Occupational Therapy. 2015;78(12):727-38. | Excluded after Title and Abstract Screening |  |
| 8 | Argentzell E, Bäckström M, Lund K, Eklund M. Exploring mediators of the recovery process over time among mental health service users, using a mixed model regression analysis based on cluster RCT data. BMC psychiatry. 2020;20(1):520. | Included |  |
| 9 | Asbjørnslett M, Berg SR, Einarsdottir V, Skarpaas LS. Stranded in the living room: A narrative study of occupational disruption and imbalance as experienced by two Norwegian students during the COVID-19 pandemic lockdown. Journal of Occupational Science. 2023;30(2):184-95. | Excluded after Title and Abstract Screening |  |
| 10 | Avrech Bar M, Shelef L, Bart O. Do participation and self-efficacy of mothers to children with ASD predict their children’s participation? Research in Autism Spectrum Disorders. 2016;24:1-10. | Excluded after Title and Abstract Screening |  |
| 11 | Backman CL. Occupational balance: exploring the relationships among daily occupations and their influence on well-being. Canadian Journal of Occupational Therapy. 2004;71(4):202-9. | Excluded after Title and Abstract Screening |  |
| 12 | Backman CL, Kennedy SM, Chalmers A, Singer J. Participation in paid and unpaid work by adults with rheumatoid arthritis. Journal of Rheumatology. 2004;31(1):47-57. | Excluded after Title and Abstract Screening |  |
| 13 | Bahadır Z, Yaran M, Özkan E, Ekici G, Huri M, Akel S. Translation, Psychometric and Concept Analysis of the Occupational Balance-Questionnaire Based on a Turkish Population. Occupational Therapy in Health Care. 2023;37(1):101-18. | Excluded after Title and Abstract Screening |  |
| 14 | Bakker K, Steultjens E, Price L. The lived experiences of adults with a visual impairment who experience fatigue when performing daily activities. British Journal of Occupational Therapy. 2019;82(8):485-92. | Excluded after Title and Abstract Screening |  |
| 15 | Barney KF, Perkinson MA. CHAPTER 30 - The future of gerontological occupational therapy. In: Barney KF, Emerita C, Perkinson MA, editors. Occupational Therapy with Aging Adults: Mosby; 2016. p. 469-88. | Excluded after Title and Abstract Screening |  |
| 16 | Bazyk S, Bazyk J. Meaning of Occupation-Based Groups for Low-Income Urban Youths Attending After-School Care. American Journal of Occupational Therapy. 2009;63(1):69-80. | Excluded after Title and Abstract Screening |  |
| 17 | Bergson K, Clarke C. Appreciating empowerment as a vital tool for creating occupational engagement...College of Occupational Therapists Conference, June 28-30, 2016. British Journal of Occupational Therapy. 2016;79:59-60. | Excluded after Title and Abstract Screening |  |
| 18 | Bilen A, Bodell S, Hook A. Unpicking occupational balance: a systematic literature review...College of Occupational Therapists Conference, June 28-30, 2016. British Journal of Occupational Therapy. 2016;79:106-7. | Excluded after Title and Abstract Screening |  |
| 19 | Binesh M, Aghili R, Mehraban AH. Occupational balance in people with type-2 diabetes: A comparative cross-sectional study. British Journal of Occupational Therapy. 2021;84(2):122-9. | Excluded after Title and Abstract Screening |  |
| 20 | Gunnarsson BA, Wagman P, Hedin K, Håkansson C. Treatment of depression and/or anxiety–outcomes of a randomised controlled trial of the tree theme method® versus regular occupational therapy. BMC psychology. 2018;6:1-10. | Included |  |
| 21 | Bonsaksen T, Lindstad MØ, Håkansson C, Wagman P, Cordier R. Rasch Analysis of the Norwegian Version of the Occupational Balance Questionnaire in a Sample of Occupational Therapy Students. Occupational Therapy International. 2021:1-11. | Excluded after Title and Abstract Screening |  |
| 22 | Borgh M, Eek F, Wagman P, Håkansson C. Organisational factors and occupational balance in working parents in Sweden. Scandinavian Journal of Occupational Therapy. 2018;46(3):409-16. | Excluded after Title and Abstract Screening |  |
| 23 | Cabrera-Martos I, Escamilla-Sevilla F, Marín-Romero B, Muñoz-Vigueras N, Rodríguez-Torres J, López-López L, et al. Balance Ability and Occupational Performance in Patients With Parkinson's Disease and On-Medication-State Freezing of Gait. Rehabilitation Nursing. 2022;47(4):138-46. | Excluded after Title and Abstract Screening |  |
| 24 | Calvo-Paz M, Guevara-Ramírez J, Zapata-López JS, Realpe-Martinez DL. Occupational balance during Covid-19 lockdown among occupational therapy academics. Brazilian Journal of Occupational Therapy / Cadernos Brasileiros de Terapia Ocupacional. 2022;30:1-16. | Excluded after Title and Abstract Screening |  |
| 25 | Cha YJ. Life satisfaction analysis between occupational balance (OB) group and occupational imbalance (OI) group. PLoS One. 2022;17(7):e0271715. | Excluded after Title and Abstract Screening |  |
| 26 | Chemen S, Gopalla YN. Lived experiences of older adults living in the community during the COVID-19 lockdown – The case of Mauritius. Journal of Aging Studies. 2021;57:100932. | Excluded after Title and Abstract Screening |  |
| 27 | Christensen JR, Konge MB, Devantier M, Brandt CJ, Gommesen JB, Kolind MI. Preferred Content in Occupational Therapy-Based Weight Loss Interventions: A Survey. OTJR: Occupation, Participation & Health. 2022;42(3):199-208. | Excluded after Title and Abstract Screening |  |
| 28 | Clouston TJ. Whose occupational balance is it anyway? The challenge of neoliberal capitalism and work–life imbalance. British Journal of Occupational Therapy. 2014;77(10):507-15. | Excluded after Title and Abstract Screening |  |
| 29 | Concheiro-Moscoso P, Groba B, Martínez-Martínez FJ, Miranda-Duro MDC, Nieto-Riveiro L, Pousada T, et al. Study for the Design of a Protocol to Assess the Impact of Stress in the Quality of Life of Workers. International Journal of Environmental Research and Public Health. 2021;18(4). | Excluded after Title and Abstract Screening |  |
| 30 | Connolly P. How occupational therapists enable single homeless people to achieve meaningful occupation. Mental Health Occupational Therapy. 2005;10(3):84-7. | Excluded after Title and Abstract Screening |  |
| 31 | Cook AM, Polgar JM. Glossary. In: Cook AM, Polgar JM, editors. Assistive Technologies (Fourth Edition). St. Louis (MO): Mosby; 2015. p. 459-70. | Excluded after Title and Abstract Screening |  |
| 32 | Crist PH, Davis CG, Coffin PS. The effects of employment and mental health status on the balance of work, play/leisure, self-care, and rest. Occupational Therapy in Mental Health. 2000;15(1):27-42. | Excluded after Title and Abstract Screening |  |
| 33 | Davy G, Barbaro J, Unwin K, Clark M, Jellett R, Date P, et al. Leisure, community, workforce participation and quality of life in primary and secondary caregivers of autistic children. Autism Research. 2024. | Excluded after Title and Abstract Screening |  |
| 34 | Davy G, Barbaro J, Unwin K, Dissanayake C. Leisure, Employment, Community Participation, and Quality of Life in Primary Caregivers of Autistic Children: A Qualitative Study. Journal of Autism and Developmental Disorders. 2023:1-13. | Excluded after Title and Abstract Screening |  |
| 35 | de Diego-Alonso C, Alegre-Ayala J, Buesa A, Blasco-Abadía J, López-Royo MP, Roldán-Pérez P, et al. Multidimensional analysis of sedentary behaviour and participation in Spanish stroke survivors (Part&Sed-Stroke): a protocol for a longitudinal multicentre study. BMJ Open. 2023;13(2):e065628. | Excluded after Title and Abstract Screening |  |
| 36 | Değerli M, Aydoner S, Altuntaş O, Bumin G. Does working style affect the occupational balance and life satisfaction of office workers in Turkey? A comparison study. Work. 2023. | Excluded after Title and Abstract Screening |  |
| 37 | Dhas BN, Carrasco R, Reinoso GA, Backman C. Occupational Balance Among Parents of Typically Developing Children and Parents of Children With Disabilities. American Journal of Occupational Therapy. 2023;77(1):1-8. | Excluded after Title and Abstract Screening |  |
| 38 | Dhas BN, Wagman P. Occupational balance from a clinical perspective. Scandinavian Journal of Occupational Therapy. 2022;29(5):373-9. | Excluded after Title and Abstract Screening |  |
| 39 | Dubouloz C, Chevrier J, Savoie-Zajc L. Transformation learning among persons with cardiac problems to achieve a balance of occupation. Canadian Journal of Occupational Therapy. 2001;68(3):171-85. | Excluded after Title and Abstract Screening |  |
| 40 | Dür M, Röschel A, Oberleitner-Leeb C, Herrmanns V, Pichler-Stachl E, Mattner B, et al. Development and validation of a self-reported questionnaire to assess occupational balance in parents of preterm infants. PLoS One. 2021;16(11):e0259648. | Excluded after Title and Abstract Screening |  |
| 41 | Dür M, Röschel A, Oberleitner-Leeb C, Herrmanns V, Pichler-Stachl E, Mattner B, et al. Associations Between Parental Occupational Balance, Subjective Health, and Clinical Characteristics of VLBW Infants. Frontiers in Pediatriacs. 2022;10:816221. | Excluded after Title and Abstract Screening |  |
| 42 | Dür M, Sadloňová M, Haider S, Binder A, Stoffer M, Coenen M, et al. Health determining concepts important to people with Crohn's disease and their coverage by patient-reported outcomes of health and wellbeing.  Journal of Crohn’s and Colitis. 2014;8(1):45-55. | Excluded after Title and Abstract Screening |  |
| 43 | Dür M, Steiner G, Stoffer MA, Fialka-Moser V, Kautzky-Willer A, Dejaco C, et al. Initial evidence for the link between activities and health: Associations between a balance of activities, functioning and serum levels of cytokines and C-reactive protein. Psychoneuroendocrinology. 2016;65:138-48. | Excluded after Title and Abstract Screening |  |
| 44 | Dür M, Steiner G, Fialka-Moser V, Kautzky-Willer A, Dejaco C, Prodinger B, et al. Development of a new occupational balance-questionnaire: incorporating the perspectives of patients and healthy people in the design of a self-reported occupational balance outcome instrument. Health & Quality of Life Outcomes. 2014;12(1):45-. | Excluded after Title and Abstract Screening |  |
| 45 | Dür M, Unger J, Stoffer M, Drăgoi R, Kautzky-Willer A, Fialka-Moser V, et al. Definitions of occupational balance and their coverage by instruments. British Journal of Occupational Therapy. 2015;78(1):4-15. | Excluded after Title and Abstract Screening |  |
| 46 | Dures E, Cramp F, Hackett K, Primdahl J. Fatigue in inflammatory arthritis. Best Practice & Research Clinical Rheumatology. 2020;34(2):101526. | Excluded after Title and Abstract Screening |  |
| 47 | Dziedzic K, Hammond A. Chapter 1 - Overview of the aims and management of rheumatological conditions: The multidisciplinary approach. In: Dziedzic K, Hammond A, editors. Rheumatology. Edinburgh: Churchill Livingstone; 2010. p. 1-15. | Excluded after Title and Abstract Screening |  |
| 48 | Edgelow M, Krupa T. Randomized Controlled Pilot Study of an Occupational Time-Use Intervention for People With Serious Mental Illness. American Journal of Occupational Therapy. 2011;65(3):267-76. | Included |  |
| 49 | Eklund M, Backstrom M. Worker role perceptions and work participation among people with mental health issues taking part in interventions focusing on everyday life. Work (Reading, Mass). 2023. | Excluded after Fulltext Review | Occupational Balance not addressed |
| 50 | Eklund M, Erlandsson L, Leufstadius C. Time Use in Relation to Valued and Satisfying Occupations among People with Persistent Mental Illness: Exploring Occupational Balance. Journal of Occupational Science. 2010;17(4):231-8. | Excluded after Title and Abstract Screening |  |
| 51 | Eklund M, Leufstadius C, Bejerholm U. Time use among people with psychiatric disabilities: implications for practice. Psychiatric Rehabilitation Journal. 2009;32(3):177-91. | Excluded after Title and Abstract Screening |  |
| 52 | Eklund M, Lund K, Argentzell E. The impact of the BEL intervention on levels of motivation, engagement and recovery in people who attend community mental health services. Scandinavian Journal of Occupational Therapy. 2023:1‐11. | Included |  |
| 53 | Eklund M, Tjornstrand C, Sandlund M, Argentzell E. Effectiveness of Balancing Everyday Life (BEL) versus standard occupational therapy for activity engagement and functioning among people with mental illness - a cluster RCT study. BMC psychiatry. 2017;17(1):363. | Included |  |
| 54 | Eklund M. Minor long-term effects 3-4 years after the ReDO™ intervention for women with stress-related disorders: A focus on sick leave rate, everyday occupations and well-being. Work. 2017;58(4):527-36. | Excluded after Fulltext Review | Study design |
| 55 | Eklund M. Occupational value outcomes among people with mental health issues participating in activity-based interventions–The importance of internal and external factors. Scandinavian Journal of Occupational Therapy. 2023:1-9. | Excluded after Fulltext Review | Occupational Balance not addressed |
| 56 | Eklund M, Argentzell E. Perception of occupational balance by people with mental illness: A new methodology. Scandinavian Journal of Occupational Therapy. 2016;23(4):304-13. | Excluded after Title and Abstract Screening |  |
| 57 | Eklund M, Brunt D, Argentzell E. Perceived occupational balance and well-being among people with mental illness living in two types of supported housing. Scandinavian Journal of Occupational Therapy. 2020;27(6):450-61. | Excluded after Title and Abstract Screening |  |
| 58 | Eklund M, Erlandsson L-K. Quality of life and client satisfaction as outcomes of the Redesigning Daily Occupations (ReDO) programme for women with stress-related disorders: A comparative study. Work. 2013;46(1):51-8. | Excluded after Fulltext Review | Occupational Balance not addressed |
| 59 | Eklund M, Erlandsson L-K. Return to Work Outcomes of the Redesigning Daily Occupations (ReDO) Program for Women with Stress-Related Disorders—A Comparative Study. Women & Health. 2011;51(7):676-92. | Excluded after Fulltext Review | Occupational Balance not addressed |
| 60 | Eklund M, Orban K, Argentzell E, Bejerholm U, Tjörnstrand C, Erlandsson L-K, Håkansson C. The linkage between patterns of daily occupations and occupational balance: Applications within occupational science and occupational therapy practice. Scandinavian Journal of Occupational Therapy. 2017;24(1):41-56. | Excluded after Title and Abstract Screening |  |
| 61 | Eklund M, Wästberg BA, Erlandsson LK. Work outcomes and their predictors in the R edesigning D aily O ccupations (Re DO) rehabilitation programme for women with stress‐related disorders. Australian Occupational Therapy Journal. 2013;60(2):85-92. | Excluded after Fulltext Review | Occupational Balance not addressed |
| 62 | Ercan Doğu S, Günal A, Pekçetin S, Örsel S, Wagman P, Håkansson C. Validity and reliability of the Turkish Occupational Balance Questionnaire (OBQ11-T) in mental health. Scandinavian Journal of Occupational Therapy. 2023;30(6):796-802. | Excluded after Title and Abstract Screening |  |
| 63 | Ercan Doğu S, Örsel S. The relationship between psychopathology, occupational balance, and quality of life among people with schizophrenia. Australian Occupational Therapy Journal. 2023;70(3):314-26. | Excluded after Title and Abstract Screening |  |
| 64 | Eriksson T, Karlström E, Jonsson H, Tham K. An exploratory study of the rehabilitation process of people with stress-related disorders. Scandinavian Journal of Occupational Therapy. 2010;17(1):29-39. | Excluded after Title and Abstract Screening |  |
| 65 | Eriksson T, Westerberg Y, Jonsson H. Experiences of women with stress-related ill health in a therapeutic gardening program. Canadian Journal of Occupational Therapy. 2011;78(5):273-81. | Excluded after Title and Abstract Screening |  |
| 66 | Espiritu EW, Yeatts PE, Evetts CL. Predictors of General Well-Being in Postprofessional Students of Occupational Science and Occupational Therapy. Open Journal of Occupational Therapy (OJOT). 2024;12(1):1-11. | Excluded after Title and Abstract Screening |  |
| 67 | Farhadian M, Akbarfahimi M, Hassani Abharian P, Khalafbeigi M, Yazdani F. The Effect of Leisure Intervention on Occupational Performance and Occupational Balance in Individuals with Substance Use Disorder: A Pilot Study. Occupational Therapy International. 2024;2024:1-8. | Included |  |
| 68 | Ferreira de Sousa I, Mendonça Magalhães FL, Seabra Castilho Simões SH, da Silva Oliveira IB, Nazareth Dias AR, Mendes Paranhos AC. Ballroom dancing as a strategy for occupational balance and stress reduction in university students in the health área. Revista Família, Ciclos de Vida e Saúde no Contexto Social (REFACS). 2022;10(2):131-42. | Included |  |
| 69 | Forhan M, Backman C. Letter to the editor. Authors' response...Forhan M. Backman C. (2010) Exploring occupational balance in adults with rheumatoid arthritis Otjr Occup Participation Health 30, 133-141. OTJR: Occupation, Participation & Health. 2010;30(3):143-. | Excluded after Title and Abstract Screening |  |
| 70 | Fox J, Erlandsson LK, Shiel A. A feasibility study of the Redesigning Daily Occupations (ReDO(TM)-10) programme in an Irish context Scandinavian Journal of Occupational Therapy. 2022;29(5):415-29. | Excluded after Fulltext Review | Study design |
| 71 | Fox J, Erlandsson L-K, McSharry J, Shiel A. How does ReDO®-10 work? Understanding the mechanisms of action of an intervention focused on daily activities and health from the perspective of participants. Evaluation and Program Planning. 2022;92:102092. | Excluded after Title and Abstract Screening |  |
| 72 | Franc I, Roberts M, Mauldin S, Rosa-Distefano G. Inter-Rater and Intra-Rater Reliability of the Kansas University Sitting Balance Scale...American Occupational Therapy Association Annual Conference & Expo, April 4-7, 2019, New Orleans, Louisiana. American Journal of Occupational Therapy. 2019;73:1-. | Excluded after Title and Abstract Screening |  |
| 73 | Friedman ZL, Banta C. A Call to Arms for Professional Sustainability and Practitioner's Mental Health. Occupational Therapy In Health Care. 2023:1-14. | Excluded after Title and Abstract Screening |  |
| 74 | Gibbs LB, Klinger L. Rest is a meaningful occupation for women with hip and knee osteoarthritis. OTJR: Occupation, Participation & Health. 2011;31(3):143-50. | Excluded after Title and Abstract Screening |  |
| 75 | Gonzalez EC, Hernandez EC, Coltrane AK, Mancera JM. The Correlation between Physical Activity and Grade Point Average for Health Science Graduate Students. OTJR: Occupation, Participation & Health. 2014;34(3):160-7. | Excluded after Title and Abstract Screening |  |
| 76 | González-Bernal JJ, Santamaría-Peláez M, González-Santos J, Rodríguez-Fernández P, León Del Barco B, Soto-Cámara R. Relationship of Forced Social Distancing and Home Confinement Derived from the COVID-19 Pandemic with the Occupational Balance of the Spanish Population. Journal of Clinical Medicine. 2020;9(11). | Excluded after Title and Abstract Screening |  |
| 77 | González-Román L, Peral-Gómez P, Garrido-Pedrosa J, Zango-Martín I, Wagman P, Sánchez-Pérez A. Occupational balance of Spanish occupational therapists - a challenge. Scandinavian Journal of Occupational Therapy. 2023;30(4):444-51. | Excluded after Title and Abstract Screening |  |
| 78 | Goodman J, Locke C. Chapter 3 - Occupations and the occupational therapy process. In: Goodman J, Hurst J, Locke C, Petruzziello V, editors. Occupational Therapy for People with Learning Disabilities. Edinburgh: Churchill Livingstone; 2009. p. 43-67. | Excluded after Title and Abstract Screening | Duplicate |
| 79 | Goodman J, Locke C. Chapter 3 - Occupations and the occupational therapy process. In: Goodman J, Hurst J, Locke C, Petruzziello V, editors. Occupational Therapy for People with Learning Disabilities. Edinburgh: Churchill Livingstone; 2009. p. 43-67. | Excluded after Title and Abstract Screening |  |
| 80 | Groezinger KL, Honsa CJ, Wilkinson DA, Simpson EK. Occupational Transitions of Family Caregivers of Loved Ones with Dementia. Internet Journal of Allied Health Sciences & Practice. 2023;21(1):1-9. | Excluded after Title and Abstract Screening |  |
| 81 | Günal A, Pekçetin S, Demirtürk F, Şenol H, Håkansson C, Wagman P. Validity and reliability of the Turkish Occupational Balance Questionnaire (OBQ11-T). Scandinavian Journal of Occupational Therapy. 2020;27(7):493-9. | Excluded after Title and Abstract Screening |  |
| 82 | Günal A, Pekçetin S, Wagman P, Håkansson C, Kayıhan H. Occupational balance and quality of life in mothers of children with cerebral palsy. British Journal of Occupational Therapy. 2022;85(1):37-43. | Excluded after Title and Abstract Screening |  |
| 83 | Güney Yılmaz G, Avcı H, Akı E. A new tool to measure occupational balance: Adolescent Occupational Balance Scale (A-OBS). Scandinavian Journal of Occupational Therapy. 2023;30(6):782-95. | Excluded after Title and Abstract Screening |  |
| 84 | Güney Yılmaz G, Zengin G, Temuçin K, Aygün D, Akı E. How the occupational balance of healthcare professionals changed in the COVID-19 pandemic: A mixed design study. Australian Occupational Therapy Journal. 2021;68(6):520-34. | Excluded after Title and Abstract Screening |  |
| 85 | Gunnarsson AB, Hakansson C, Hedin K, Wagman P. Outcomes of the Tree Theme Method versus regular occupational therapy: A longitudinal follow-up. Australian Occupational Therapy Journal. 2022;69(4):379-90. | Included |  |
| 86 | Guszkowska M, Dąbrowska-Zimakowska A. Occupational balance, changes in occupations and psychological well-being of university students during the COVID-19 pandemic. Scandinavian Journal of Occupational Therapy. 2023;30(4):463-74. | Excluded after Title and Abstract Screening |  |
| 87 | Hagelskjaer V, Nielsen KT, von Bulow C, Oestergaard LG, Graff M, Waehrens EE. Evaluating a complex intervention addressing ability to perform activities of daily living among persons with chronic conditions: study protocol for a randomised controlled trial (ABLE). BMJ open. 2021;11(11):e051722. | Excluded after Fulltext Review | Study protocol |
| 88 | Haines AJ, Mackenzie L, Honey A, Middleton PG. Occupations and balance during the transition to motherhood with a lifetime chronic illness: A scoping review examining cystic fibrosis, asthma, and Type-1 diabetes. Australian Occupational Therapy Journal. 2023;70(6):730-44. | Excluded after Title and Abstract Screening |  |
| 89 | Håkansson C, Ahlborg G, Jr. Occupations, perceived stress, and stress-related disorders among women and men in the public sector in Sweden. Scandinavian Journal of Occupational Therapy. 2017;24(1):10-7. | Excluded after Title and Abstract Screening |  |
| 90 | Håkansson C, Lexén A. The combination of psychosocial working conditions, occupational balance and sociodemographic characteristics and their associations with no or negligible stress symptoms among Swedish occupational therapists - a cross-sectional study. BMC Health Services Research. 2021;21(1):471. | Excluded after Title and Abstract Screening |  |
| 91 | Håkansson C, Lissner L, Bjorkelund C, Sonn U. Engagement in patterns of daily occupations and perceived health among women of working age. Scandinavian Journal of Occupational Therapy. 2009;16(2):110-7. | Excluded after Title and Abstract Screening |  |
| 92 | Håkansson C, Ahlborg G. Occupational imbalance and the role of perceived stress in predicting stress-related disorders. Scandinavian Journal of Occupational Therapy. 2018;25(4):278-87. | Excluded after Title and Abstract Screening |  |
| 93 | Håkansson C, Björkelund C, Eklund M. Associations between women's subjective perceptions of daily occupations and life satisfaction, and the role of perceived control. Australian Occupational Therapy Journal. 2011;58(6):397-404. | Excluded after Title and Abstract Screening |  |
| 94 | Håkansson C, Gunnarsson AB, Wagman P. Occupational balance and satisfaction with daily occupations in persons with depression or anxiety disorders. Journal of Occupational Science. 2023;30(2):196-202. | Included |  |
| 95 | Håkansson C, Leo U, Oudin A, Arvidsson I, Nilsson K, Österberg K, Persson R. Organizational and social work environment factors, occupational balance and no or negligible stress symptoms among Swedish principals - a cross-sectional study. BMC Public Health. 2021;21(1):1-9. | Excluded after Title and Abstract Screening |  |
| 96 | Håkansson C, Lexén A. Work conditions as predictors of Swedish occupational therapists' occupational balance. Scandinavian Journal of Occupational Therapy. 2023;30(4):520-6. | Excluded after Title and Abstract Screening |  |
| 97 | Håkansson C, Milevi S, Eek F, Oudin A, Wagman P. Occupational balance, work and life satisfaction in working cohabiting parents in Sweden. Scandinavian Journal of Public Health. 2019;47(3):366-74. | Excluded after Title and Abstract Screening |  |
| 98 | Håkansson C, Wagman P, Hagell P. Construct validity of a revised version of the Occupational Balance Questionnaire. Scandinavian Journal of Occupational Therapy. 2020;27(6):441-9. | Excluded after Title and Abstract Screening |  |
| 99 | Hammond A. What is the role of the occupational therapist? Best Practice & Research Clinical Rheumatology. 2004;18(4):491-505. | Excluded after Title and Abstract Screening |  |
| 100 | Hansen AØ, Boll M, Skaarup L, Hansen T, Dür M, Stamm T, Kristensen HK. Danish translation and validation of the Occupational Balance Questionnaire. Scandinavian Journal of Occupational Therapy. 2022;29(5):380-94. | Excluded after Title and Abstract Screening |  |
| 101 | Healing K, Lowrie D. Exploring the occupational experiences of livestock farmers during drought: A narrative inquiry. Australian Journal of Rural Health. 2023;31(5):855-65. | Excluded after Title and Abstract Screening |  |
| 102 | Hearle D, Prince J, Rees V. An exploration of the relationship between place of residence, balance of occupation and self-concept in older adults as reflected in life narratives. Quality in Ageing. 2005;6(4):24-33. | Excluded after Title and Abstract Screening |  |
| 103 | Hearle D, Rees V, Prince J. Balance of occupation in older adults: experiences in a residential care home. Quality in Ageing & Older Adults. 2012;13(2):125-34. | Excluded after Title and Abstract Screening |  |
| 104 | Hernandez R, Schneider S, Wagman P, Håkansson C, Spruijt-Metz D, Pyatak EA. Validity and Reliability of the Occupational Balance Questionnaire (OBQ11) in a U.S. Sample of Adults With Type 1 Diabetes. American Journal of Occupational Therapy. 2023;77(4):1-11. | Excluded after Title and Abstract Screening |  |
| 105 | Hernandez R, Vidmar A, Pyatak EA. Lifestyle balance, restful and strenuous occupations, and physiological activation. Journal of Occupational Science. 2020;27(4):547-62. | Excluded after Title and Abstract Screening |  |
| 106 | Hersche R, Weise A, Della Bella S, Michel G, Marco M, Kool J. Feasibility and Preliminary Results of a Short Inpatient Energy-Management Education for Person with MS-fatigue. Archives of physical medicine and rehabilitation. 2019;100(10):e77. | Excluded after Fulltext Review | Article not peer-reviewed |
| 107 | Hersche R, Weise A, Michel G, Kesselring J, Bella SD, Barbero M, Kool J. Three-week inpatient energy management education (IEME) for persons with multiple sclerosis-related fatigue: Feasibility of a randomized clinical trial. Multiple Sclerosis and Related Disorders. 2019;35:26-33. | Excluded after Fulltext Review | Occupational Balance not addressed |
| 108 | Hersche R, Roser K, Weise A, Michel G, Barbero M. Fatigue self-management education in persons with disease-related fatigue: A comprehensive review of the effectiveness on fatigue and quality of life. Patient Education and Counseling. 2022;105(6):1362-78. | Excluded after Title and Abstract Screening |  |
| 109 | Ho ECM, Dür M, Stamm T, Siu AMH. Measuring the occupational balance of people with insomnia in a Chinese population: Preliminary psychometric evidence on the Chinese version of the Occupational Balance Questionnaire. Hong Kong Journal of Occupational Therapy. 2020;33(2):33-41. | Excluded after Title and Abstract Screening |  |
| 110 | Ho ECM, Siu AMH. Occupational Therapy Practice in Sleep Management: A Review of Conceptual Models and Research Evidence. Occupational Therapy International. 2018:1-12. | Excluded after Fulltext Review | Study design |
| 111 | Ho ECM, Siu AMH. Evaluation of an occupation-based sleep programme for people with Insomnia. Hong Kong Journal of Occupational Therapy. 2022;35(2):168-79. | Included |  |
| 112 | Hodgetts S, McConnell D, Zwaigenbaum L, Nicholas D. The Impact of Autism Services on Mothers' Occupational Balance and Participation. OTJR: Occupation, Participation & Health. 2014;34(2):81-93. | Excluded after Title and Abstract Screening |  |
| 113 | Hogan LM, Björklund Carlstedt A, Wagman P. Occupational therapy and stress-related exhaustion - a scoping review. Scandinavian Journal of Occupational Therapy. 2023;30(7):1047-63. | Excluded after Title and Abstract Screening |  |
| 114 | Holmefur M, Lidström-Holmqvist K, Roshanay AH, Arvidsson P, White S, Janeslätt G. Pilot Study of Let's Get Organized: A Group Intervention for Improving Time Management. American Journal of Occupational Therapy. 2019;73(5):1-10. | Included |  |
| 115 | Holmefur M, Roshanay A, White S, Janeslätt G, Vimefall E, Lidström-Holmqvist K. Evaluation of the "Let's Get Organized" group intervention to improve time management: protocol for a multi-centre randomised controlled trial. Trials. 2021;22(1):1-15. | Excluded after Fulltext Review | Trial registration |
| 116 | Honoré H, Boll ML, Hansen AØ, Kristensen HK. Putting occupational balance on the radar: Content validity of the 11-item Danish Occupational Balance Questionnaire. British Journal of Occupational Therapy. 2024;87(3):169-80. | Excluded after Title and Abstract Screening |  |
| 117 | Hovbrandt P, Carlsson G, Nilsson K, Albin M, Håkansson C. Occupational balance as described by older workers over the age of 65. Journal of Occupational Science. 2019;26(1):40-52. | Excluded after Title and Abstract Screening |  |
| 118 | Howes ML, Ellison D. Understanding the occupational identity of care-givers for people with mental health problems. British Journal of Occupational Therapy. 2022;85(4):274-82. | Excluded after Title and Abstract Screening |  |
| 119 | Hultqvist J, Lund K, Argentzell E, Eklund M. Predictors of clinically important improvements in occupational and quality of life outcomes among mental health service users after completion and follow-up of a lifestyle intervention: multiple regression modelling based on longitudinal data. BMC Psychol. 2019;7(1):83. | Included |  |
| 120 | Hurst J. Chapter 5 - Occupation and health promotion. In: Goodman J, Hurst J, Locke C, Petruzziello V, editors. Occupational Therapy for People with Learning Disabilities. Edinburgh: Churchill Livingstone; 2009. p. 85-98. | Excluded after Title and Abstract Screening |  |
| 121 | Trial registration: Irct20120910010806N. leisure and substance use disorders. https://trialsearchwhoint/Trial2aspx?TrialID=IRCT20120910010806N11. 2023. | Excluded after Fulltext Review | Trial registration |
| 122 | Trial registration: Irct20180105038224N. Designing Occupation-Centered Self-management interventions and investigating its effect on Performance, Satisfaction and self- efficacy of people diagnosed with type 2 diabetes. https://trialsearchwhoint/Trial2aspx?TrialID=IRCT20180105038224N1. 2018. | Excluded after Fulltext Review | Trial registration |
| 123 | Trial registration: Irct20220630055330N. The effects of group-based education of handling skills for parents with children with cerebral palsy on family empowerment and child’s functional mobility. https://trialsearchwhoint/Trial2aspx?TrialID=IRCT20220630055330N1. 2022. | Excluded after Title and Abstract Screening |  |
| 124 | Iwama MK. Chapter 8 - Applying the Kawa Model: Comprehending Occupation in Context. In: Iwama MK, editor. The Kawa Model. Edinburgh: Churchill Livingstone; 2006. p. 157-76. | Excluded after Title and Abstract Screening |  |
| 125 | Jakobsen K, Magnus E, Lundgren S, Reidunsdatter RJ. Everyday life in breast cancer survivors experiencing challenges: A qualitative study. Scandinavian Journal of Occupational Therapy. 2018;25(4):298-307. | Excluded after Title and Abstract Screening |  |
| 126 | Jessen-Winge C, Kolind M, Lee K, Leth MB, Surrow S, Knürr DS, et al. Occupational balance as a component in weight loss interventions. Scandinavian Journal of Occupational Therapy. 2023;30(7):1028-36. | Excluded after Title and Abstract Screening |  |
| 127 | Jessen-Winge C, Enemark Larsen A, Solgaard Nielsen S, Reffstrup Christensen J. Five Vital Components of an OT-Inspired Municipal Weight Loss Program Obtained Through Research Circles...American Occupational Therapy Association (AOTA) INSPIRE 2021 (Virtual), April 6-30, 2021. American Journal of Occupational Therapy. 2021;75:1-. | Excluded after Title and Abstract Screening |  |
| 128 | Jo YJ, Kim H. Effects of the model of human occupation-based home modifications on the time use, occupational participation and activity limitation in people with disabilities: a pilot randomized controlled trial. Disability and Rehabilitation-Assistive Technology. 2022;17(2):127-33. | Excluded after Fulltext Review | Occupational Balance not addressed |
| 129 | Johnsen AM, Theodorsson E, Broström A, Wagman P, Fransson EI. Work-related factors and hair cortisol concentrations among men and women in emergency medical services in Sweden. Scientific Reports. 2023;13(1):12877. | Excluded after Title and Abstract Screening |  |
| 130 | Jonsson H, Borell L, Sadlo G. Retirement: an occupational transition with consequences for temporality, balance and meaning of occupations. Journal of Occupational Science. 2000;7(1):29-37. | Excluded after Title and Abstract Screening |  |
| 131 | Jonsson H, Persson D. Towards an experiential model of occupational balance: an alternative perspective on flow theory analysis. Journal of Occupational Science. 2006;13(1):62-73. | Excluded after Title and Abstract Screening |  |
| 132 | Jung JH, Ko JY, Hong I, Jung MY, Park JH. Effects of a time-use intervention in isolated patients with coronavirus disease 2019: A randomized controlled study. PloS one. 2023;18(6):e0287118. | Included |  |
| 133 | Jung JH, Won JJ, Ko JY. Psychological rehabilitation for isolated patients with COVID-19 infection: a randomized controlled study. PloS one. 2022;17(12):e0278475. | Excluded after Title and Abstract Screening |  |
| 134 | Karlsson L, Erlandsson LK, Cregård A, Nordgren L, Lydell M. Taking control of one's everyday life - a qualitative study of experiences described by participants in an occupational intervention. BMC Public Health. 2023;23(1):605. | Excluded after Title and Abstract Screening |  |
| 135 | Karlsson L, Ivarsson A, Erlandsson L-K. Exploring risk factors for developing occupational ill health – departing from an occupational perspective. Scandinavian Journal of Occupational Therapy. 2022;29(5):363-72. | Excluded after Title and Abstract Screening |  |
| 136 | Kassberg A-C, Nyman A, Larsson Lund M. Perceived occupational balance in people with stroke. Disability & Rehabilitation. 2021;43(4):553-8. | Excluded after Title and Abstract Screening |  |
| 137 | Trial registration: Kct. Effects of time use intervention on occupational balance, mental health and quality of life in COVID-19 patients. https://trialsearchwhoint/Trial2aspx?TrialID=KCT0005711. 2020. | Excluded after Fulltext Review | Trial registration |
| 138 | Kersten ML, Coxon K, Lee H, Wilson NJ. Traversing the community is uncertain, socially complex and exhausting: Autistic youth describe experiences of travelling to participate in their communities. Journal of Transport & Health. 2020;18:100922. | Excluded after Title and Abstract Screening |  |
| 139 | Kirsh B, Martin L, Hultqvist J, Eklund M. Occupational Therapy Interventions in Mental Health: A Literature Review in Search of Evidence. Occupational Therapy in Mental Health. 2019;35(2):109-56. | Excluded after Fulltext Review | Study design |
| 140 | Koome F, Hocking C, Sutton D. Why Routines Matter: The Nature and Meaning of Family Routines in the Context of Adolescent Mental Illness. Journal of Occupational Science. 2012;19(4):312-25. | Excluded after Title and Abstract Screening |  |
| 141 | Kos D, Nijs J, Meirte J, Willekens B, Nagels G, D'Hooghe MB. The effectiveness of a self-management occupational therapy intervention on activity performance in persons with MS-related fatigue: a randomized clinical trial. (#60). Multiple sclerosis (Houndmills, Basingstoke, England). 2012;18(5):S46‐S7. | Excluded after Fulltext Review | Article not peer-reviewed |
| 142 | Kos D, Ferdinand S, Duportail M, Eijssen I, Schouteden S, Kerkhofs L, et al. Assessing life balance of European people with multiple sclerosis: A multicenter clinimetric study within the RIMS network. Multiple Sclerosis and Related Disorders. 2020;39:101879. | Excluded after Title and Abstract Screening |  |
| 143 | Lee CD, Kim MY, Foster E. The Relationship Between Occupational Balance and Wellbeing in Older Adults: Time-Use Perspective. Archives of Physical Medicine and Rehabilitation. 2019;100(10):e133. | Excluded after Title and Abstract Screening |  |
| 144 | Lee SC, Awan M, Chaudhary U, John P. Occupational Disruptions Among Health Professional Faculty During COVID-19 Pandemic. Open Journal of Occupational Therapy (OJOT). 2022;10(3):1-9. | Excluded after Title and Abstract Screening |  |
| 145 | Leenders JMP, Geurts ACH, Steultjens EMJ, Packer TL, Cup EHC. Test-retest reliability of three life balance measures in people with neuromuscular disease: the activity card sort-NL, the activity calculator, and the occupational balance questionnaire. Disability & Rehabilitation. 2023:1-7. | Excluded after Title and Abstract Screening |  |
| 146 | Lexén A, Kåhlin I, Erlandsson LK, Håkansson C. Occupational Health among Swedish Occupational Therapists: A Cross-Sectional Study. International Journal of Environmental Research and Public Health. 2020;17(10). | Excluded after Title and Abstract Screening |  |
| 147 | Lieb LC. Occupational Therapy in an Ecological Context: Ethics and Practice. American Journal of Occupational Therapy. 2022;76(3):1-4. | Excluded after Title and Abstract Screening |  |
| 148 | Lindmark U, Ahlstrand I, Ekman A, Berg L, Hedén L, Källstrand J, et al. Health-promoting factors in higher education for a sustainable working life - protocol for a multicenter longitudinal study. BMC Public Health. 2020;20(1):233. | Excluded after Title and Abstract Screening |  |
| 149 | Locke C. Chapter 8 - Leisure. In: Goodman J, Hurst J, Locke C, Petruzziello V, editors. Occupational Therapy for People with Learning Disabilities. Edinburgh: Churchill Livingstone; 2009. p. 135-45. | Excluded after Title and Abstract Screening |  |
| 150 | Lozano-Lozano M, Galiano-Castillo N, Gonzalez-Santos A, Ortiz-Comino L, Sampedro-Pilegaard M, Martín-Martín L, Arroyo-Morales M. Effect of mHealth plus occupational therapy on cognitive function, mood and physical function in people after cancer: Secondary analysis of a randomized controlled trial. Annals of Physical and Rehabilitation Medicine. 2023;66(2):101681. | Excluded after Title and Abstract Screening |  |
| 151 | Luchynsky MK, Ashbaugh K, Bowser A, Campisi E, Gleixner M, Heinbach B, Snak A. Efficacy of Utilizing the Group Mode of Treatment Delivery in OT for Skilled Nursing Facility Settings...American Occupational Therapy Association INSPIRE Annual Conference & Expo, April 20-23, 2023, Kansas City, Missouri. American Journal of Occupational Therapy. 2023;77:1-. | Excluded after Title and Abstract Screening |  |
| 152 | Lundqvist LO, Ivarsson AB, Rask M, Brunt D, Schröder A. The attendees' view of quality in community-based day centre services for people with psychiatric disabilities. Scandinavian Journal of Occupational Therapy. 2018;25(3):162-71. | Excluded after Title and Abstract Screening |  |
| 153 | M F. Occupational Balance: Who decides? And what happens when it goes wrong?...RCOT (Royal College of Occupational Therapist) Annual Conference 2017. British Journal of Occupational Therapy. 2017;80:46-7. | Excluded after Title and Abstract Screening |  |
| 154 | Madsen CMT, Christensen JR, Bremander A, Primdahl J. Perceived challenges at work and need for professional support among people with inflammatory arthritis - A qualitative interview study. Scandinavian Journal of Occupational Therapy. 2023;30(5):640-9. | Excluded after Title and Abstract Screening |  |
| 155 | Magnusson L, Håkansson C, Brandt S, Oberg M, Orban K. Occupational balance and sleep among women. Scandinavian Journal of Occupational Therapy. 2021;28(8):643-51. | Excluded after Title and Abstract Screening |  |
| 156 | Mahdizadeh A, Khankeh H, Ghodsi H, Hosseini SA, Akbarfahimi N. Post-COVID-19 survivors' strategies for improving occupational balance: A qualitative study. British Journal of Occupational Therapy. 2023;86(11):777-86. | Excluded after Title and Abstract Screening |  |
| 157 | Matuska K, Bass J. Life Balance and Stress in Adults With Medical Conditions or Obesity. OTJR (Thorofare N J). 2016;36(2):74-81. | Excluded after Title and Abstract Screening |  |
| 158 | McGuire BK, Crowe TK, Law M, VanLeit B. Mothers of children with disabilities: occupational concerns and solutions. OTJR: Occupation, Participation & Health. 2004;24(2):54-63. | Excluded after Title and Abstract Screening |  |
| 159 | Milton Y, Roe S. Occupational therapy home programmes for children with unilateral cerebral palsy using bimanual and modified constraint induced movement therapies: A critical review. British Journal of Occupational Therapy. 2017;80(6):337-49. | Excluded after Title and Abstract Screening |  |
| 160 | Morville AL, Wagman P, Håkansson C. A Rasch analysis of the Danish version of the occupational balance questionnaire (OBQ11). Scandinavian Journal of Occupational Therapy. 2024;31(1):2327356. | Excluded after Title and Abstract Screening |  |
| 161 | Mthembu TG, Brown Z, Cupido A, Razak G, Wassung D. Family caregivers' perceptions and experiences regarding caring for older adults with chronic diseases. South African Journal of Occupational Therapy. 2016;46(1):83-8. | Excluded after Title and Abstract Screening |  |
| 162 | N Dhas B, Wagman P, Marji FA, Håkansson C, Carrasco R. Translation and initial validation of the occupational balance questionnaire to Arabic—Occupational Balance Questionnaire-A. British Journal of Occupational Therapy. 2022;85(7):533-40. | Excluded after Title and Abstract Screening |  |
| 163 | Naidoo D, Gurayah T, Kharva N, Stott T, Trend SJ, Mamane T, Mtolo S. Having a child with cancer: African mothers' perspective. South African Journal of Occupational Therapy. 2016;46(3):49-54. | Excluded after Title and Abstract Screening |  |
| 164 | Trial registration: Nct. Balancing Everyday Life - A Lifestyle Intervention for People With Psychiatric Disorders. https://clinicaltrialsgov/show/NCT02619318. 2015. | Excluded after Fulltext Review | Study protocol |
| 165 | Trial registration: Nct. Managing Fatigue in People With Parkinson's Disease. https://clinicaltrialsgov/show/NCT04267107. 2020. | Excluded after Fulltext Review | Trial registration |
| 166 | Trial registration: Nct. Mindfulness in University Students. ATENEU Program. https://clinicaltrialsgov/show/NCT05929430. 2023. | Excluded after Fulltext Review | Trial registration |
| 167 | Trial registration: Nct. OcupApp: occupational Self-analysis Intervention Through an Mobile Application. https://clinicaltrialsgov/show/NCT05867823. 2023. | Excluded after Fulltext Review | Study protocol |
| 168 | Trial registration: Nct. Examining the Effects of Activity Management in Women With Fibromyalgia Syndrome. https://clinicaltrialsgov/show/NCT05821036. 2023. | Excluded after Fulltext Review | Study protocol |
| 169 | Trial registration: Nct. Investigation of the Effects of Temporal Adaptation Approach in Post-Discharge Stroke Individuals. https://clinicaltrialsgov/ct2/show/NCT06085469. 2023. | Excluded after Fulltext Review | Study protocol |
| 170 | Trial registration: Nct. Online Occupational Therapy on Occupational Balance, Well-being and Quality of Life in Syrian Refugee Children. https://clinicaltrialsgov/show/NCT05233345. 2022. | Excluded after Title and Abstract Screening |  |
| 171 | Trial registration: Nct. "Let's Get Organized" in Adult Psychiatric/Habilitation Care. https://clinicaltrialsgov/show/NCT03654248. 2018. | Excluded after Title and Abstract Screening |  |
| 172 | Nielsen SS, Skou ST, Larsen AE, Polianskis R, Arendt-Nielsen L, Østergaard AS, et al. Changes in pain, daily occupations, lifestyle, and health following an occupational therapy lifestyle intervention: a secondary analysis from a feasibility study in patients with chronic high-impact pain. Scandinavian Journal of Pain. 2024;24(1). | Included |  |
| 173 | Nielsen SS, Christensen JR, Søndergaard J, Mogensen VO, Enemark Larsen A, Skou ST, Simonÿ C. Feasibility assessment of an occupational therapy lifestyle intervention added to multidisciplinary chronic pain treatment at a Danish pain centre: a qualitative evaluation from the perspectives of patients and clinicians. International Journal of Qualitative Studies on Health & Well-Being. 2021;16(1):1-13. | Excluded after Title and Abstract Screening |  |
| 174 | Nissmark S, Malmgren Fänge A. Occupational balance among family members of people in palliative care. Scandinavian Journal of Occupational Therapy. 2020;27(7):500-6. | Excluded after Title and Abstract Screening |  |
| 175 | Norberg EB, Boman K, Löfgren B, Brännström M. Occupational performance and strategies for managing daily life among the elderly with heart failure. Scandinavian Journal of Occupational Therapy. 2014;21(5):392-9. | Excluded after Title and Abstract Screening |  |
| 176 | Trial registration: Ntr. Life Balance Study. https://trialsearchwhoint/Trial2aspx?TrialID=NTR7231. 2018. | Excluded after Fulltext Review | Trial registration |
| 177 | Nyman A, Kassberg A-C, Lund ML. Perceived occupational value in people with acquired brain injury. Scandinavian Journal of Occupational Therapy. 2021;28(5):391-8. | Excluded after Title and Abstract Screening |  |
| 178 | Olsson A, Erlandsson L-K, Håkansson C. The occupation-based intervention REDO™-10: Long-term impact on work ability for women at risk for or on sick leave. Scandinavian Journal of Occupational Therapy. 2020;27(1):47-55. | Included |  |
| 179 | Ortiz-Rubio A, Cabrera-Martos I, Haro-Piedra E, López-López L, Rodríguez-Torres J, Granados-Santiago M, Valenza MC. Exploring perceived occupational balance in women with fibromyalgia. A descriptive study. Scandinavian Journal of Occupational Therapy. 2022;29(5):395-402. | Excluded after Title and Abstract Screening |  |
| 180 | Palmer K, D'Angelo S, Clare Harris E, Linaker C, Coggon D. 0066Problems of vision, hearing and balance and risks of workplace injury. Occupational & Environmental Medicine. 2014;71:A7-A. | Excluded after Title and Abstract Screening |  |
| 181 | Peral-Gómez P, López-Roig S, Pastor-Mira M, Abad-Navarro E, Valera-Gran D, Håkansson C, Wagman P. Cultural Adaptation and Psychometric Properties of the Spanish Version of the Occupational Balance Questionnaire: An Instrument for Occupation-Based Research. International Journal of Environmental Research and Public Health. 2021;18(14). | Excluded after Fulltext Review | Study design |
| 182 | Park MO, Lee JH. Role Value, Occupational Balance, and Quality of Life: A Cross-Sectional Study on Exploring the Urban Older People Perspective in South Korea. International Journal of Environmental Research and Public Health. 2022;19(5). | Excluded after Title and Abstract Screening |  |
| 183 | Park S, Lee HJ, Jeon BJ, Yoo EY, Kim JB, Park JH. Effects of occupational balance on subjective health, quality of life, and health-related variables in community-dwelling older adults: A structural equation modeling approach. PLoS One. 2021;16(2):e0246887. | Excluded after Title and Abstract Screening |  |
| 184 | Park S, Park J-H. Causal Effects of Occupational Balance (OB) on Participation, Health, Quality of Life, and Stress With People in Adulthood...American Occupational Therapy Association Annual Conference & Expo, April 4-7, 2019, New Orleans, Louisiana. American Journal of Occupational Therapy. 2019;73:1-. | Excluded after Title and Abstract Screening |  |
| 185 | Park S, Park J-H, Lee HJ, Jeon B-J, Yoo EY, Kim JB. Effects of Occupational Balance (OB) on Health, Quality of Life and Related Variables in Community-Dwelling Older Adults: Structural Equation Modeling...2020 AOTA Annual Conference & Expo. American Journal of Occupational Therapy. 2020;74:1-. | Excluded after Title and Abstract Screening |  |
| 186 | Parry R, Jones E. Chapter 13 - More than having a say – user participation in learning disability services. In: Goodman J, Hurst J, Locke C, Petruzziello V, editors. Occupational Therapy for People with Learning Disabilities. Edinburgh: Churchill Livingstone; 2009. p. 197-224. | Excluded after Title and Abstract Screening |  |
| 187 | Parsonage J, Naylor Lund K, Dawes H, Almoajil H, Eklund M. An exploration of occupational choices in adolescence: A constructivist grounded theory study. Scandinavian Journal of Occupational Therapy. 2022;29(6):464-81. | Excluded after Title and Abstract Screening |  |
| 188 | Patt N, Kupjetz M, Kool J, Hersche R, Oberste M, Joisten N, et al. Effects of inpatient energy management education and high-intensity interval training on health-related quality of life in persons with multiple sclerosis: A randomized controlled superiority trial with six-month follow-up. Multiple Sclerosis and Related Disorders. 2023;78:104929. | Excluded after Title and Abstract Screening |  |
| 189 | Pekçetin E, Ekici G, Çetinkaya M, Pehlivan F, Torpil B, Pekçetin S. Comparisons of Occupational Balance Within Informal Caregivers of Individuals With Schizophrenia. OTJR (Thorofare N J). 2023:15394492231202416. | Excluded after Title and Abstract Screening |  |
| 190 | Pekçetin S, Günal A. Effect of Web-Based Time-Use Intervention on Occupational Balance During the Covid-19 Pandemic. Canadian Journal of Occupational Therapy. 2021;88(1):83-90. | Included |  |
| 191 | Pentland W, McColl MA. Occupational integrity: another perspective on 'life balance'. Canadian Journal of Occupational Therapy. 2008;75(3):135-8. | Excluded after Title and Abstract Screening |  |
| 192 | Peral-Gómez P, Espinosa-Sempere C, Navarrete-Muñoz EM, Hurtado-Pomares M, Juárez-Leal I, Valera-Gran D, Sánchez-Pérez A. The Spanish version of Occupational Balance Questionnaire: psychometric properties and normative data in a representative sample of adults. Ann Med. 2022;54(1):3211-8. | Excluded after Title and Abstract Screening |  |
| 193 | Peral-Gómez P, López-Roig S, Pastor-Mira M, Abad-Navarro E, Valera-Gran D, Håkansson C, Wagman P. Cultural Adaptation and Psychometric Properties of the Spanish Version of the Occupational Balance Questionnaire: An Instrument for Occupation-Based Research. International Journal of Environmental Research and Public Health. 2021;18(14). | Excluded after Title and Abstract Screening |  |
| 194 | Pettican A, Prior S. 'It's a new way of life': an exploration of the occupational transition of retirement. British Journal of Occupational Therapy. 2011;74(1):12-9. | Excluded after Title and Abstract Screening |  |
| 195 | Peyton CG, Huang Y-h, Syväoja K, Lohman H. Chapter 5 - Aging Well: Health Promotion and Disease Prevention. In: Lohman HL, Byers-Connon S, Padilla RL, editors. Occupational Therapy with Elders (Fourth Edition). St. Louis (MO): Mosby; 2019. p. 51-68. | Excluded after Title and Abstract Screening |  |
| 196 | Pilegaard MS, Timm H, Birkemose HK, Dupont SB, Joergensen DS, la Cour K. A resource-oriented intervention addressing balance in everyday activities and quality of life in people with advanced cancer: protocol for a feasibility study. Pilot Feasibility Stud. 2022;8(1):86. | Excluded after Title and Abstract Screening |  |
| 197 | Ramos R, Röschel A, Crevenna R, Jordakieva G, Andrews MR, Dür M, Stamm T. Occupational Balance and Depressive Symptoms During the COVID-19 Pandemic: A Four-Wave Panel Study on the Role of Daily Activities in Austria. Journal of Occupational & Environmental Medicine. 2022;64(8):694-8. | Excluded after Title and Abstract Screening |  |
| 198 | Ranehov L, Håkansson C. Mothers' experiences of their work as healthcare assistants for their chronic disabled child. Scandinavian Journal of Occupational Therapy. 2019;26(2):121-34. | Excluded after Title and Abstract Screening |  |
| 199 | Raveica G, Raveica IC, Ciucurel MM. Occupational Balance in Children of 8-10 Years and its Influence on School Performance. Procedia - Social and Behavioral Sciences. 2012;46:3752-6. | Excluded after Title and Abstract Screening |  |
| 200 | Raya-Ruiz MA, Rodríguez-Bailón M, Castaño-Monsalve B, Vidaña-Moya L, Fernández-Solano AJ, Merchán-Baeza JA. Study protocol for a non-randomised controlled trial: community-based occupational therapy intervention on mental health for people with acquired brain injury (COT-MHABI). PloS one. 2022;17(10):e0274193. | Excluded after Title and Abstract Screening |  |
| 201 | Reeck N. Betätigungsbalance von Kindern und Jugendlichen in Zeiten der Corona-Pandemie/Occupational balance of children and adolescents in the times of the Corona pandemic. WFOT Bulletin. 2021;77(2):124-30. | Excluded after Title and Abstract Screening |  |
| 202 | Rodríguez-Fernández P, González-Santos J, Santamaría-Peláez M, Soto-Cámara R, González-Bernal JJ. Exploring the Occupational Balance of Young Adults during Social Distancing Measures in the COVID-19 Pandemic. International Journal of Environmental Research and Public Health. 2021;18(11). | Excluded after Title and Abstract Screening |  |
| 203 | Rodríguez-Rivas C, Camacho-Montaño LR, García-Bravo C, García-de-Miguel M, Pérez-de-Heredia-Torres M, Huertas-Hoyas E. Effects of Social Isolation Measures Caused by the COVID-19 Pandemic on Occupational Balance, Participation, and Activities' Satisfaction in the Spanish Population. International Journal of Environmental Research and Public Health. 2022;19(11). | Excluded after Title and Abstract Screening |  |
| 204 | Romero-Ayuso DM, Toledano-González A, Pinilla-Cerezo M, Sánchez-Rodríguez Ó, García-Arenas JJ, Triviño-Juárez JM, Ortíz-Rubio A. Occupational Balance and Emotional Regulation in People With and Without Serious Mental Illness. Canadian Journal of Occupational Therapy. 2024;91(1):100-9. | Excluded after Title and Abstract Screening |  |
| 205 | Romero-Ayuso D, García-López R, Lozano-Villena C, Martínez JR, Parga-Amado P, García-Ferreiro P, et al. Usability of a mobile phone application to enhance activities of daily living in occupational therapy services for breast cancer survivors. Hong Kong Journal of Occupational Therapy. 2023;36(2):128-40. | Excluded after Fulltext Review | Study design |
| 206 | Romero-Tébar A, Rodríguez-Hernández M, Segura-Fragoso A, Cantero-Garlito PA. Analysis of Occupational Balance and Its Relation to Problematic Internet Use in University Occupational Therapy Students. Healthcare (Basel). 2021;9(2). | Excluded after Title and Abstract Screening |  |
| 207 | Röschel A, Wagner C, Dür M. Associations between occupational balance, subjective health, and well-being of informal caregivers of older persons based on a cross-sectional study. BMC Geriatrics. 2022;22(1):445. | Excluded after Title and Abstract Screening |  |
| 208 | Röschel A, Wagner C, Dür M. Examination of validity, reliability, and interpretability of a self-reported questionnaire on Occupational Balance in Informal Caregivers (OBI-Care) - A Rasch analysis. PLoS One. 2021;16(12):e0261815. | Excluded after Title and Abstract Screening |  |
| 209 | Rothacker-Peyton S, Wilson-Anderson K, Mayer K. Nursing Faculty Professional Quality of Life and Resilience in a Changing Educational Environment. Journal of Nursing Education. 2022;61(6):330-3. | Excluded after Title and Abstract Screening |  |
| 210 | Ryan D, Naughton M, de Faoite M, Dowd T, Morrissey AM. An Occupation-Based Lifestyle Lecture Intervention as Part of Inpatient Addiction Recovery Treatment: Exploring Occupational Performance, Balance and Personal Recovery. Substance Abuse. 2023;17:11782218231165123. | Included |  |
| 211 | Vas S, Vajaratkar P Understanding the Occupational Balance in Young Working Mothers in Goa, India: A Cross Sectional Study...59th Annual National Conference of All India Occupational Therapists' Association (OTICON 2022), March 11-13, 2022 (virtual). Indian Journal of Occupational Therapy (Wolters Kluwer India Pvt Ltd). 2022;54(2):62-3. | Excluded after Title and Abstract Screening |  |
| 212 | Sahu S, Bandyopadhyay B, Chandrashekaran M, Mohapatra D, David KQ, Ghanta S, Arya S. Quality of Life and Occupational Balance during Corona Virus Disease 2019 Lockdown in India: A Survey on Parents of Children below 16 Years. Indian Journal of Occupational Therapy (Wolters Kluwer India Pvt Ltd). 2021;53(4):138-43. | Excluded after Title and Abstract Screening |  |
| 213 | Salazar BL. Supporting the Paternal Role and Transition Home From the NICU: A Mixed Method Study. Open Journal of Occupational Therapy (OJOT). 2022;10(2):1-19. | Excluded after Title and Abstract Screening |  |
| 214 | Salehi S, Seyed S, Raji P, Nakhostin Ansari N, Håkansson C, Wagman P. Translation, cultural adaptation and psychometric properties of the Persian version of Occupational Balance Questionnaire in healthy adults. British Journal of Occupational Therapy. 2023;86(8):587-94. | Excluded after Title and Abstract Screening |  |
| 215 | Sánchez-Pérez A, Mendialdua-Canales D, Hurtado-Pomares M, Peral-Gómez P, Juárez-Leal I, Espinosa-Sempere C, et al. The ATENción Plena en Enfermedad de Alzheimer (ATENEA-Mindfulness in Alzheimer's Disease) Program for Caregivers: study Protocol for a Randomized Controlled Trial. Healthcare (Basel, Switzerland). 2022;10(3). | Excluded after Title and Abstract Screening |  |
| 216 | Sandqvist G, Eklund M. Daily occupations--performance, satisfaction and time use, and relations with well-being in women with limited systemic sclerosis. Disability & Rehabilitation. 2008;30(1):27-35. | Excluded after Title and Abstract Screening |  |
| 217 | Shaker M. Preventing burnout through wellness and an attitude of gratitude. Annals of Allergy, Asthma & Immunology. 2021;126(3):215-6. | Excluded after Title and Abstract Screening |  |
| 218 | Shochat I, Lipskaya-Velikovsky L, Hadas Lidor N. Cultural Adaptation of Action Over Inertia, a Participation-oriented Intervention for Individuals with Serious Mental Illness. Israel Journal of Occupational Therapy / Ketab ’et Yiśreʼeliy Leriypẇy be ’Iysẇq. 2020;29(1):E23-E. | Excluded after Title and Abstract Screening |  |
| 219 | Singh G, Srivastava M, Srivastava M, Srivastava RK, Srivastava P. Work-Life Balance and Occupational Stress of the Non-Gazetted Central Reserve Police Force Jawans in Chandauli. Indian Journal of Community Health. 2023;35(3):324-8. | Excluded after Title and Abstract Screening |  |
| 220 | Stamm T, Binder A, Prodinger B. Letter to the editor...Forhan M, Backman C. (2010) Exploring occupational balance in adults with rheumatoid arthritis Otjr Occup Participation Health 30, 133-141. OTJR: Occupation, Participation & Health. 2010;30(3):142-. | Excluded after Title and Abstract Screening |  |
| 221 | Stamm T, Lovelock L, Stew G, Nell V, Smolen J, Machold K, et al. I have a disease but I am not ill: a narrative study of occupational balance in people with rheumatoid arthritis. OTJR: Occupation, Participation & Health. 2009;29(1):32-9. | Excluded after Title and Abstract Screening |  |
| 222 | Stamm T, Wright J, Machold K, Sadlo G, Smolen J. Occupational balance of women with rheumatoid arthritis: a qualitative study. Musculoskeletal Care. 2004;2(2):101-12. | Excluded after Title and Abstract Screening |  |
| 223 | Sturesson M, Edlund C, Falkdal AH, Bernspång B. Healthcare encounters and return to work: a qualitative study on sick-listed patients’ experiences. Primary Health Care Research & Development. 2014;15(4):464-75. | Excluded after Title and Abstract Screening |  |
| 224 | Su Lin Yong A, Price L. The human occupational impact of partner and close family caregiving in dementia: a meta-synthesis of the qualitative research, using a bespoke quality appraisal tool. British Journal of Occupational Therapy. 2014;77(8):410-21. | Excluded after Title and Abstract Screening |  |
| 225 | Tapia V, Isralowitz EB, Deng K, Nguyen NT, Young M, Como DH, et al. Exploratory analysis of college students' occupational engagement during COVID-19. Journal of Occupational Science. 2022;29(4):545-61. | Excluded after Title and Abstract Screening |  |
| 226 | Temizkan E, Köse B, Şahin S. Social determinants of life satisfaction in emerging adults. Children and Youth Services Review. 2023;151:107050. | Excluded after Title and Abstract Screening |  |
| 227 | To-Miles F, Håkansson C, Wagman P, Backman CL. Exploring the Associations Among Occupational Balance and Health of Adults With and Without Inflammatory Arthritis. Arthritis Care Res (Hoboken). 2022;74(1):22-30. | Excluded after Title and Abstract Screening |  |
| 228 | Tse T, Roberts E, Garvie J, Sutton E, Munro A. The impact of COVID‐19 restrictions on occupational balance: A mixed method study of the experience of Australian occupational therapists. Australian Occupational Therapy Journal. 2022;69(1):89-97. | Excluded after Title and Abstract Screening |  |
| 229 | Uhrmann L, Hovengen I, Wagman P, Håkansson C, Bonsaksen T. The Norwegian Occupational Balance Questionnaire (OBQ11-N) – development and pilot study. Scandinavian Journal of Occupational Therapy. 2019;26(7):546-51. | Excluded after Title and Abstract Screening |  |
| 230 | Uthede S, Nilsson I, Wagman P, Håkansson C, Farias L. Occupational balance in parents of pre-school children: Potential differences between mothers and fathers. Scandinavian Journal of Occupational Therapy. 2023;30(8):1199-208. | Excluded after Title and Abstract Screening |  |
| 231 | VanLeit B, Crowe TK. Outcomes of an occupational therapy program for mothers of children with disabilities: impact on satisfaction with time use and occupational performance. American Journal of Occupational Therapy. 2002;56(4):402-10. | Excluded after Fulltext Review | Occupational Balance not addressed |
| 232 | Vidaña-Moya L, Eklund M, Merchán-Baeza JA, Peral-Gómez P, Zango-Martín I, Hultqvist J. Cross-Cultural Adaptation, Validation and Reliability of the Spanish Satisfaction with Daily Occupations-Occupational Balance (SDO-OB): An Evaluation Tool for People with Mental Disorders. International Journal of Environmental Research and Public Health. 2020;17(23). | Excluded after Title and Abstract Screening |  |
| 233 | Wagman P, Nordin M, Alfredsson L, Westerholm PJ, Fransson EI. Domestic work division and satisfaction in cohabiting adults: Associations with life satisfaction and self-rated health. Scandinavian Journal of Occupational Therapy. 2017;24(1):24-31. | Excluded after Title and Abstract Screening |  |
| 234 | Wagman P, Ahlstrand I, Björk M, Håkansson C. Occupational balance and its association with life satisfaction in men and women with rheumatoid arthritis. Musculoskeletal Care. 2020;18(2):187-94. | Excluded after Title and Abstract Screening |  |
| 235 | Wagman P, Gunnarsson AB, Hjärthag F, Hedin K, Håkansson C. Quality of life, sense of coherence and occupational balance one year after an occupational therapy intervention for people with depression and anxiety disorders. Work. 2023;76(2):561-8. | Included |  |
| 236 | Wagman P, Håkansson C. Exploring occupational balance in adults in Sweden. Scandinavian Journal of Occupational Therapy. 2014;21(6):415-20. | Excluded after Title and Abstract Screening |  |
| 237 | Wagman P, Håkansson C. Introducing the Occupational Balance Questionnaire (OBQ). Scandinavian Journal of Occupational Therapy. 2014;21(3):227-31. | Excluded after Title and Abstract Screening |  |
| 238 | Wagman P, Håkansson C. Occupational balance from the interpersonal perspective: A scoping review. Journal of Occupational Science. 2019;26(4):537-45. | Excluded after Title and Abstract Screening |  |
| 239 | Wagman P, Håkansson C, Björklund A. Occupational balance as used in occupational therapy: A concept analysis. Scandinavian Journal of Occupational Therapy. 2012;19(4):322-7. | Excluded after Title and Abstract Screening |  |
| 240 | Wagman P, Håkansson C, Jacobsson C, Falkmer T, Björklund A. What is considered important for life balance? Similarities and differences among some working adults. Scandinavian Journal of Occupational Therapy. 2012;19(4):377-84. | Excluded after Title and Abstract Screening |  |
| 241 | Wagman P, Hjärthag F, Håkansson C, Hedin K, Gunnarsson AB. Factors associated with higher occupational balance in people with anxiety and/or depression who require occupational therapy treatment. Scandinavian Journal of Occupational Therapy. 2021;28(6):426-32. | Excluded after Title and Abstract Screening |  |
| 242 | Wagman P, Lindmark U, Rolander B, Wåhlin C, Håkansson C. Occupational balance in health professionals in Sweden. Scandinavian Journal of Occupational Therapy. 2017;24(1):18-23. | Excluded after Title and Abstract Screening |  |
| 243 | Watford P, Jewell V, Atler K. Increasing Meaningful Occupation for Women Who Provide Care for Their Spouse: A Pilot Study. OTJR (Thorofare N J). 2019;39(4):213-21. | Excluded after Fulltext Review | Study design |
| 244 | Welford J, McKenna C. Postural tachycardia syndrome: a UK occupational therapy perspective. British Journal of Cardiology. 2016;23(4):1-5. | Excluded after Title and Abstract Screening |  |
| 245 | Wheeler A, Bloch E, Blaylock S, Root J, Ibanez K, Newman K, et al. Delirium education for family caregivers of patients in the intensive care unit: A pilot study. PEC Innovation. 2023;2:100156. | Excluded after Title and Abstract Screening |  |
| 246 | Willis Boslego LA, Munterfering Phillips CE, Atler KE, Tracy BL, Van Puymbroeck M, Schmid AA. Impact of yoga on balance, balance confidence and occupational performance for adults with diabetic peripheral neuropathy: A pilot study. British Journal of Occupational Therapy. 2017;80(3):155-62. | Excluded after Fulltext Review | Occupational Balance not addressed |
| 247 | Wingren M, Lidström-Holmqvist K, Roshanai AH, Arvidsson P, Janeslätt G, White S, Holmefur M. One-year follow-up after the time management group intervention Let's Get Organized. Scandinavian Journal of Occupational Therapy. 2022;29(4):305-14. | Excluded after Fulltext Review | Occupational Balance not addressed |
| 248 | Yazdani F, Bonsaksen T. Introduction to the Model of Occupational Wholeness. Ergoscience. 2017;12(1):32-6. | Excluded after Title and Abstract Screening |  |
| 249 | Yazdani F, Harb A, Rassafiani M, Nobakht L, Yazdani N. Occupational therapists’ perception of the concept of occupational balance. Scandinavian Journal of Occupational Therapy. 2018;25(4):288-97. | Excluded after Title and Abstract Screening |  |
| 250 | Yazdani F, Roberts D, Yazdani N, Rassafiani M. Occupational balance: A study of the sociocultural perspective of Iranian occupational therapists. Canadian Journal of Occupational Therapy. 2016;83(1):53-62. | Excluded after Title and Abstract Screening |  |
| 251 | Yu Y, Manku M, Backman CL. Measuring occupational balance and its relationship to perceived stress and health. Canadian Journal of Occupational Therapy. 2018;85(2):117-27. | Excluded after Title and Abstract Screening | Duplicate |
| 252 | Yu Y, Manku M, Backman CL. Measuring occupational balance and its relationship to perceived stress and health. Canadian Journal of Occupational Therapy. 2018;85(2):117-27. | Excluded after Title and Abstract Screening |  |
| 253 | Yucel H. Relationship between playing-related factors and occupational balance in professional violinists. Work. 2023. | Excluded after Title and Abstract Screening |  |
| 254 | Zhao F, Friedman PH, Toussaint L, Webb JR, Freedom J. Translation and validation of the Chinese version of the Friedman life balance scale among nursing students: A psychometric analysis. Nurse Education in Practice. 2023;66:103505. | Excluded after Title and Abstract Screening |  |
| 255 | Zhu C, Shen JH, Lee C-C, Liu S. Does religion belief matter to self-employment of rural elderly? Evidence from China. Journal of Asian Economics. 2022;83:101548. | Excluded after Title and Abstract Screening |  |
| 256 | Zurich I. Occupational rehabilitation: restoring work confidence for long-term unemployed people. British Journal of Occupational Therapy. 2016;79:54-. | Excluded after Fulltext Review | Article not peer-reviewed |
