# Supplementary material for: Interventions promoting occupational balance in adults: A systematic literature review
Source: PLoS One. 2025 Jun 5;20(6):e0325061. doi: 10.1371/journal.pone.0325061 (PMC12176295; doi:10.1371/journal.pone.0325061)
Supplement: Attachment — Submitted filename: Review PLoS dec-24.docx [file pone.0325061.s007.docx]

Thank you for the opportunity to read and review this manuscript, titled Interventions promoting Occupational Balance in Adults: A Systematic Literature Review. The authors have used a setup of methodologies that seem relevant to a systematic literature review and leave a thorough audit trail of their work process, from registration of the protocol in PROSPERO to the current manuscript. It is an ambitious review, performed with attention to details. Still, there are some problems to be solved and the paper cannot be accepted in its present form.

1. Neither the abstract nor the introduction presents the rationale for the study. Why is the review needed? Actually, this is best expression in the discussion, page 22, line 341 and the following, but it needs to be stated in the intro – and abstract – as well.
2. Page 3, lines 54-58. This sentence is unclear and needs to be reformulated.
3. Under heading ‘Gap of knowledge…’, page 5, lines 111 and forward should include the rationale, but instead there is a reasoning that does not seem totally logical. It is true that occupational balance is seen as a dynamic phenomenon, but is it therefore a good idea to “examine occupational balance interventions in populations with different ages, socio-economic situations, diagnoses, and regional backgrounds as well as in different contexts and settings (e.g., health care, health prevention, social service)”? Please rewrite this section for clarity.
4. The research questions are clearly formulated, but I am not sure how they align with the results/discussion/conclusion. Please see comments below related to the results.
5. The methods are mainly thoroughly and clearly presented, but I have a few comments:
   a. Page 6, lines 131 and the following: Two of the inclusion criteria have been assigned with digit 2.
   b. Same paragraph, you indicate that the publications should be in English to be included, but om the following page (page 7, line 149) you write that also articles in German were included. Please include German already on page 6.
   c. Page 10, last paragraph: I was wondering why you change tense from imperfect to present tense. Is it to indicate that the first explanations are from the literature reviewed and the last two (in present tense) were stipulated by you authors? Please clarify.
6. Results
   a. Please see the below table for comments regarding alignment between research questions and the results.

| **Research questions** | **Results** | **Comment** |
| --- | --- | --- |
| 1) What interventions exist that address occupational balance in adults? | - Prisma flow chart - Study characteristics - Assessment of methodological quality - Interventions promoting occupational balance | You should somewhere indicate how you arrived at these four themes/sections  Perhaps also be more detailed in the research questions? |
| 2) How effective are existing interventions in promoting adults’ occupational balance? | - Effectiveness of interventions targeting occupational balance | The result section is mostly about the instruments used.  A little is said about the pre-post studies but almost nothing about the results from the RCT studies, which I find remarkable. |

b. Besides, I think you have miscalculated the number of participants in the RCTs addressing the TTM and the BEL. Some of the publications are from the same respective RCT.
c. Page 22, line 339. The 16-week BEL is mentioned in the section, but you write that the duration of the interventions was 8-12 weeks. (Should be given in digits since the highest value is >10.) Please clarify.

7. Discussion
   a. Thank you for the study rationale given here, page 22.
   b. Page 23, line 372: Do refs 94 and 95 say what you state here? Or are they examples of multifaceted interventions? Or are they just examples of specific interventions?
   c. The discussion is very long and includes some interesting points, especially pages 26-27. But the text tends to escape form the findings, and sometimes resembles of a policy document. I suggest you review the text, shorten it, and delete your own opinions about things.
8. Conclusion: The first paragraph here seems based on the findings. But how do the second and third ones build on your findings? I think they should be substituted with the gist of what you found in relation to research question #2. The fourth paragraph is about needs for further research, thus okay.
9. References: There is a bit of a mess here, and I just give a few examples:
   a. The format is not correct. There are several examples of capitalized article titles, and there is a mix of abbreviated and non-abbreviated journal names.
   b. Some sources appear with more than one numbering. For example, ##40, 62, and 78 are the same source. Similarly, ##63 and 79 are the same. This is just something I noticed *en passant*, so there may be more errors of this kind.
10. Language and typos
    a. I do not have English as my first language, but I find the language problematic, particularly in the abstract and the introduction. So I suggest language editing.
    b. Abstract, line 16: A digit is missing in the first year stated.
    c. Page 4, line 96: The correct name of the intervention is Balancing Everyday Life. Please delete ‘of’.
    d. Page 10, line 221: Please round off 19.60 years to the nearest whole number.

My conclusion is that the paper addresses an interesting topic of relevance for researchers and staff involved in activity-based rehabilitation. There are several issues that need to be addressed, however, and although the manuscript has potential it is not suitable for publication in its present form. I believe the authors have the ability to rework the manuscript according to the comments above encourage them to resubmit a revised version.
