# Supplementary material for: Interventions promoting occupational balance in adults: A systematic literature review
Source: PLoS One. 2025 Jun 5;20(6):e0325061. doi: 10.1371/journal.pone.0325061 (PMC12176295; doi:10.1371/journal.pone.0325061)
Supplement: Attachment — Submitted filename: Response to Reviewers.docx [file pone.0325061.s009.docx]

**Krems, 20.02.2025**

**Dear Reviewers and dear Editor,**

Thank you for your valuable feedback on our manuscript. We appreciate the time and effort you have taken to review our work. We have thoroughly implemented all your comments and believe that your input has significantly improved the manuscript. Below, we have addressed each of your comments in detail.

**Reviewer 1:**

1. **Comment:** Abstract. This does not seem to be written with the same level of care and language as the rest of the manuscript. Some phrasing is difficult to understand, and the definition of OB is challenging. The results are quite non-specific, and the conclusion is a bit vague on details as to whether this is important or not.
   **Response:** We are incredibly grateful for this important comment. Based on this feedback, we have spent further time on improving the abstract and very much hope that this latest version more strongly emphasizes the relevance and clinical significance of our systematic literature review. (lines 2ff, page 1&2)
2. **Comment:** Line 80 ref (23) Is this the right one?
   **Response:** Thank you for pointing this out. We have added the correct reference (line 78, page 4).
3. **Comment:** L 81 past tense – potentially affect
   **Response:** Thank you for noticing the tense mistake, we have changed it accordingly (line 79, page 4).
4. **Comment:** L105 have (not has)
   **Response:** Thank you for raising this grammar mistake. (line 100, page 5)
5. **Comment:** L106 Don’t start sentence with Especially. The sentence is unclear in its meaning.
   **Response**: Thank you for making us aware of the lack of clarity. We fully agree with reviewer 1 that this sentence seemed out of context and have hence deleted it.
6. **Comment:** L111-115: Context getting a bit mixed
   **Response:** Thank you for raising this problem. We have accordingly changed the whole paragraph and hope that it is more targeted now. (lines 103ff, page 5)
7. **Comment:** L 116-118 need rewording. Quite confusing.
   **Response:** Thank you for making us aware of this incongruency. We have now reformulated the gap of knowledge which has led us to delete this sentence.
8. **Comment:** L121-123: research questions don’t align with clinical context mentioned earlier.
   **Response:** Thank you for this comment and for making us aware of this. We have further elaborated the contexts and added *“in diverse contexts”* in line 111, page 5. We hope that there is congruence now.
9. **Comment:** Selection criteria: Not sure why point two was added, if it didn’t matter? Is this maybe a data extraction item?
   **Response:** Thank you for bringing this up. Our intention was to emphasize that both people with and without a diagnosis were included in this review. We have now changed the formulation to make it clearer that we *“involved persons with and without any diagnoses”* (lines 122&123, page 6).
10. **Comment:** L143: Why were the databases searched over a 2-month period, instead of all on the same day, to make sure you got consistent reports from the databases?
    **Response:** Thank you for making us aware of this potential confusion. We mentioned the time frame for the entire search process, including the development of the search string, pilot searches and hand searches. The final search was carried out *on April 8^th^, 2024*. We have added this date now to the manuscript (line 134, page 6).
11. **Comment:** L149 – why searched for in German if only English language was included (L138)?
    **Response:** Thanks for pointing this out. We forgot to mention this inclusion criterion here because we noticed that there were no German hits. We have now corrected this in the manuscript, added *German* to the selection criteria in lines 127&128, and stated in the “Study characteristics” section (line 211, page 9) that *“The language of publication for all studies in the review was English”*.
12. **Comment:** L 155 – what was deemed a completely irrelevant record and who made that decision?
    **Response:** We used this to capture records that did not comply with the inclusion criteria. To enhance clarity, we removed “completely irrelevant records” (line 146, page 7).
13. **Comment:** L168-173: Can this info please be added to the supplementary material with the questions.
    **Response:** Thank you for your suggestion. We have removed the information about the NHLBI quality assessment tools in this section and instead added it in the Supplementary Material as recommended (S5 table).
14. **Comment:** L160 & 175: What was the value of using the kappa statistic? Was further discussion entailed if too low between raters?
    **Response:** Thank you for this input. We have better described the reasoning behind this in the method sections, lines 149f, page 7*: “Cohen’s kappa coefficient was calculated for the screening process to evaluate the inter-rater agreement (44) and to initiate further discussion if the coefficient was too low.*“ and added it accordingly in line 205, page 9: “*further discussion about inclusion was not necessary.”*
15. **Comment:** Study flow chart: No mention of the number of duplicates found and the number of irrelevant records.
    **Response:** Thank you for pointing this out. We adjusted the flow chart (fig 1), mentioning the number of duplicates. To avoid misunderstanding we are no longer using the expression “irrelevant records”.
16. **Comment:** Selection criteria did not say that protocols were not accepted – is this because it is assumed that no values were available for the outcomes measures?
    **Response:** Thank you for highlighting this. Our aim was to identify existing interventions that have already been tested, with findings published in peer-reviewed journals; therefore, we excluded study protocols. We have added examples to the exclusion criteria, to be more explicit on this point: “*a) were not published in a German or English-language, peer-reviewed journal (e.g., study protocols, poster presentations)”* (lines 128ff, page 6)
17. **Comment:** L199 – the reasons given for exclusion don’t seem to match what is presented in the diagram?
    **Response:** Thank you for pointing this out. To be more explicit about the reasons for exclusion at each stage of the selection process, we have added *“Records excluded based on the inclusion and exclusion criteria”* in Fig 1 in the screening phase. We also added information about the reasons for exclusion at the eligibility stage, consistent with those stated in Fig 1, in the main text at lines 199ff, page 9: *“Studies were excluded at this step because they were study protocols (n=5), trial registrations (n=7), incorrect study design (n=7) or non-peer-reviewed articles (n=4), or because occupational balance was not addressed (n=10).”*
18. **Comment:** L221: Mean age??
    **Response:** Thanks for pointing out this grammar mistake, we have changed “mean ages” to “mean age”, line 217, page 9.
19. **Comment:** L221: years since diagnosis: this isn’t in the extraction criteria? And earlier said inclusion could be with and without a condition?
    **Response:** Thank you for making us aware of this inconsistency, since we included persons with and without diagnosis we added “*if applicable*” to make this clearer, also adding “*if applicable: years since diagnosis*” in line 166, page 7 under “data extraction and data analysis”.
20. **Comment:** L225: This makes sense of the random clinical mention in introduction now. Can this important information, including the definitions be moved to the introduction and/or methods. This appears to come out of nowhere.
    **Response:** Thank you for this comment. We have now mentioned the different contexts of interventions earlier in the background section and further explained the definitions of contexts in the method section, see lines 169ff, page 8: “*For this review, study settings were categorized into clinical, community-based and academic setting. The term clinical was used to refer to medical work relating to the examination and treatment of persons based on their health status, also involving rehabilitation, as the process of returning to a way of life after being ill (46). The notion community-based was defined as a setting that takes place locally, where an individual works, plays, and performs other daily activities (47). Academic settings were related to schools, colleges, universities or connected with studying (46).”*
21. **Comment:** Table 1: How are the studies organised in the table? They don’t seem to be alphabetical, and pre-posts are before the RCTs? Found it at the end of the table -> do you mean the intervention is alphabetical? How does this relate to the narrative synthesis approach taken described in the methods?
    **Response:** Thank you for raising this question. We have added the heading *“Intervention*” to the title of the first column and included the titles of interventions accordingly. Also, we have organized the publications by study design (starting with RCTs, followed by pre-post studies, and finally cohort studies) and then alphabetically. To enhance clarity, we have also revised the explanation in the notes: *“Note. Studies are listed by study design and then alphabetically”,* see pages 11-14.
22. **Comment:** T1: Time/length of intervention not always reported or reported as missing.
    **Response:** Thank you for making us aware that the reader might search for this information in table 1. Since the focus of this table is on the characteristics of the study, we kept the information on length of intervention in table 3 “*Interventions promoting occupational balance in adults*”, page 17&18. To clarify for the reader, we added “*Additional information on the interventions reported is summarized in table 3.”* in lines 223f, page 10 in the body text and *“Additional information on the interventions is reported in table 3”* in the note for table 1, page 14.
23. **Comment:** T1: Farhadian: is it possible to conduct an analysis on 9 people? Does sample size calculation stand up?
    **Response:** Thank you for bringing up this legitimate issue. To answer research question 1, we did not exclude studies based on the results of the critical appraisal, as described in lines 236ff, page 15. However, when answering research question 2, we do indeed discuss the problem related to such a small sample size. Based on your feedback, we have put stronger emphasizes on this, beginning from line 429, page 25f *“…because the measurement instruments, study designs, and quality of the studies vary widely, some of the results must be viewed with caution. For instance, one study (66) showed significant improvements in occupational balance from pre- to post-intervention, but due to the small sample size of only nine participants the results should be treated with care.”*
24. **Comment:** T1: How many of the Swedish studies are analyses from the same cohort, performed over different time periods or relating to different outcomes? If any, can they be synthesised so as not to give the impression they are different populations.
    **Response:** Thank you for pointing this out. We tried to clarify this misunderstanding by pointing out that they are analyses from the same cohort both in table 1, page 11: *“analyses from the same study cohort as in (37)*” as well as in the body text, line 288, page 20.
25. **Comment:** Can Table 2 be colour coded or another visual method use to show the differences between the ROB categories? Can headings also be added to show which studies are the same design, instead of a,b and c. Also, consider using N/A instead of empty cells, to show that this was not applicable, rather than forgotten.
    **Response:** Thank you for this suggestion, we have implemented a colour code to show different ROB categories and inserted headings for the study designs. Also, we inserted “n/a” instead of leaving empty cells to avoid misunderstandings, table 2 on page 15&16.
26. **Comment:** Table 3: does the a with the accent indicate a mean value? Or approximately?
    **Response:** Thank you for raising this issue. We have now used a slash to indicate the duration per session to enhance readability and avoid misunderstandings, e.g. *“1hr/session”*, see table 3 on page17&18.
27. **Comment:** Heading on L286 is followed by a paragraph about how the outcomes were measured. The followed by whether results were positive or not, but not the extent of the change. Difficult to assess whether change was clinically significant.
    **Response:** Thank you for this comment. Based on your feedback, we have changed the order and content of this section, starting from line 284 on page20.
28. **Comment:** In results, strength of correlations are not documented in paragraph starting 319.
    **Response:** We added an explanation that strength of correlations were not calculated in this review (as few studies have actually calculated these calculations.). The previously discussed topic of possible correlations was changed into a more cautiously expression: *“Due to the variability of data and measurement tools across studies, it was decided not to conduct a meta-analysis and therefore no correlations were calculated or reported in this systematic review.”*, (line 330ff, page 22).
29. **Comment:** Where are the drop-out rates listed?
    **Response:** Thank you for raising this issue. We have added all drop-out rates in table 1, pages 11ff.
30. **Comment:** In discussion, the extent of change, and whether it is of any clinical significance, is not discussed?
    **Response:** Thank you for this valuable information. We have added this accordingly and now discuss the clinical significance in more detail in the section starting on line 449ff, page 26.
31. **Comment:** Paragraph starting L346: can this be reworded to address the contents of the paragraph, instead of a reference back to the results.
    **Response:** Thank you for your comment. We edited this section to avoid referring back to the results (lines 356ff, page 23): *“The interventions included in this review shared several common characteristics: they were largely delivered in person, tended to target persons with mental health problems, and employed a variety of methods to promote occupational balance and support participants in transferring these changes to their daily lives.”.*
32. **Comment:** I am missing the bit about where an improvement in OB for these populations meant something to their overall health? What was the target score for these populations?
    **Response:** Thank you for pointing this out. We added more content on this in the paragraph starting on line 449, page 26: “*As clinical significance refers to the extent to which an intervention makes a tangible and meaningful difference in the daily lives of patients or those with whom they interact (84, 85), it is arguable that not only the quantifiable changes in occupational balance measures are worth mentioning, but also subsequent benefits in other aspects of life. For example, improved occupational balance also reduced drug craving and enhanced leisure participation in individuals with substance use disorders (66). In isolated patients, improved occupational balance led to better scores in mental health and quality of life (67). Additionally, improved outcomes affected work ability in women with depression (61).”.*

**Reviewer 2:**

1. **Comment:**  Neither the abstract nor the introduction presents the rationale for the study. Why is the review needed? Actually, this is best expression in the discussion, page 22, line 341 and the following, but it needs to be stated in the intro – and abstract – as well.
   **Response:** Thank you for raising this important issue. We have now more strongly emphasized the rationale for this work both in the background and the abstract, see abstract and line 98ff, page 5.
2. **Comment:**  Page 3, lines 54-58. This sentence is unclear and needs to be reformulated.
   **Response:** Thank you for pointing this out. We have reformulated the sentence accordingly: “*Various factors can contribute to an imbalance in daily occupations. These include lack of time to complete desired or necessary tasks, limited opportunities to manage how time is allocated across activities, inflexibility, a mismatch between desired and required activities, and having either too much or too little to do.”,* lines 51ff, page3.
3. **Comment:**  Under heading ‘Gap of knowledge…’, page 5, lines 111 and forward should include the rationale, but instead there is a reasoning that does not seem totally logical. It is true that occupational balance is seen as a dynamic phenomenon, but is it therefore a good idea to “examine occupational balance interventions in populations with different ages, socio-economic situations, diagnoses, and regional backgrounds as well as in different contexts and settings (e.g., health care, health prevention, social service)”? Please rewrite this section for clarity.
   **Response:** Thank you for bringing this up. We have revised and reformulated this paragraph accordingly, see lines 101ff, page 5: “*Thus far, no systematic literature review has been conducted to synthesize and assess peer-reviewed publications analyzing interventions that address occupational balance among diverse adult populations and across various health-prevention contexts. These contexts include clinical, community-based, and academic contexts, and encompass populations of different ages, socio-economic situations, diagnoses, and regional backgrounds. Given the significant impact of occupational balance on health, it is crucial to elevate the concept to a public health priority effectively. This approach not only supports the health of the population but also aligns with societal and economic goals of maintaining a healthy population.”.*
4. **Comment:**  The research questions are clearly formulated, but I am not sure how they align with the results/discussion/conclusion. Please see comments below related to the results.
   **Response:**  Thank you for raising this critical issue. We made several adaptations in the results, discussion and conclusion sections and hope that the reasoning is more stringent now. For example the section “Effectiveness of interventions targeting occupational balance” in the discussion has been thoroughly revised (lines 426ff, page 25f).
5. **Comment:**  The methods are mainly thoroughly and clearly presented, but I have a few comments:
   a. Page 6, lines 131 and the following: Two of the inclusion criteria have been assigned with digit 2.
      **Response:** Thank you for raising this mistake. We have now implemented a list with letters for better readability, see lines 122ff on page6.
   b. Same paragraph, you indicate that the publications should be in English to be included, but om the following page (page 7, line 149) you write that also articles in German were included. Please include German already on page 6.
      **Response:**  Thanks for pointing this out. We forgot to mention this inclusion criterion here because we noticed that there were no German hits. We have now corrected this in the manuscript, added German to the inclusion criteria in lines 127&128 on page 6, and stated in the “Study characteristics” section (line 211, page 9) that *“The language of publication for all studies in the review was English”*.
   c. Page 10, last paragraph: I was wondering why you change tense from imperfect to present tense. Is it to indicate that the first explanations are from the literature reviewed and the last two (in present tense) were stipulated by you authors? Please clarify.
      **Response:** Thank you for spotting this tense mistake. Based on your feedback, we have now checked for tense mistakes throughout the manuscript.
6. **Comment:**  Results
   a. Please see the below table for comments regarding alignment between research questions and the results.

      **Response:** Thank you for this valuable table. We have changed the order of this section accordingly. First, we have added an introducing sentence to explain the structure of the section: *“The presentation of the results compromises an overview of the study characteristics, and the quality assessed, a brief description of the interventions and their effectiveness. In line with the two research questions the results are structured into the presentation of interventions addressing occupational balance, and an appraisal of their effectiveness.”* on page 8, lines 188ff. Second, we have more strongly highlighted evidence hierarchies throughout the manuscript based on study design, starting with RCTs, followed by pre-post studies and finally observational studies (see line 286ff on page 20f, table 1 on pages 11ff, and table 2 on page 15).
   b. Besides, I think you have miscalculated the number of participants in the RCTs addressing the TTM and the BEL. Some of the publications are from the same respective RCT.
      **Response:** Thank you for identifying this mistake. We have corrected this accordingly in line 214, page 9 to *“total of 641 study participants”*, added corresponding notes in table 1, pages 11ff (“*Analyses from the same study cohort as in (37)”* and “*Analyses from the same study cohort as in (57)”)* and included respective comments in the results section (line 288, page 20 *“…with all analyses based on the same study population”* and line 302, page 21 *“All four analyses were based on the same population…”*).
   c. Page 22, line 339. The 16-week BEL is mentioned in the section, but you write that the duration of the interventions was 8-12 weeks. (Should be given in digits since the highest value is >10.) Please clarify.
      **Response:** Thank you for bringing up this inconsistency. We have changed the maximum duration to 16 weeks in lines 348f, page 22 and accordingly adapted the duration of the BEL intervention in table 3, page 17f.
7. **Comment:**  Discussion
   a. Thank you for the study rationale given here, page 22.
      **Response:** Thanks for this positive remark. We have emphasized the rationale for this work in the background (lines 99ff, page 5) and abstract.
   b. Page 23, line 372: Do refs 94 and 95 say what you state here? Or are they examples of multifaceted interventions? Or are they just examples of specific interventions?
      **Response:** Thank you for making us aware of this error, unintentionally produced by our reference manager. Due to the shortening of the discussion, we deleted this part though.
   c. The discussion is very long and includes some interesting points, especially pages 26-27. But the text tends to escape form the findings, and sometimes resembles of a policy document. I suggest you review the text, shorten it, and delete your own opinions about things.
      **Response:** Thank you for this comment. We revised the discussion and discussed the actual findings more in-depth, for example social relations (lines 359ff on page 23), modes and settings of treatment (lines 369ff, page 23) or interventions that could promote occupational balance (lines 396ff, page 24).
8. **Comment**: Conclusion: The first paragraph here seems based on the findings. But how do the second and third ones build on your findings? I think they should be substituted with the gist of what you found in relation to research question #2. The fourth paragraph is about needs for further research, thus okay.
   **Response:** Thank you for this comment. We have reformulated the conclusion section, formulating a more stringent connection to research question 2 (lines 531ff, page 29).
9. **Comment:**  References: There is a bit of a mess here, and I just give a few examples:
   a. The format is not correct. There are several examples of capitalized article titles, and there is a mix of abbreviated and non-abbreviated journal names.
   b. Some sources appear with more than one numbering. For example, ##40, 62, and 78 are the same source. Similarly, ##63 and 79 are the same. This is just something I noticed *en passant*, so there may be more errors of this kind.
      **Response:** Thank you for bringing up these important issues. We have carefully reviewed and corrected the bibliography.
10. **Comment:**  Language and typos
    a. I do not have English as my first language, but I find the language problematic, particularly in the abstract and the introduction. So I suggest language editing.
       **Response:** Thank you for the important advice. We have worked through the manuscript and involved a native speaker to improve the linguistic quality, especially the abstract and introduction.
    b. Abstract, line 16: A digit is missing in the first year stated.
       **Response:** Thank you for bringing this to our attention. We have added the correct year.
    c. Page 4, line 96: The correct name of the intervention is Balancing Everyday Life. Please delete ‘of’.
       **Response:** Thank you for raising this mistake, we have corrected the name accordingly to the actual name of the intervention, line 93, page 4.
    d. Page 10, line 221: Please round off 19.60 years to the nearest whole number.
       **Response:** Thank you for bringing up this mistake, we have rounded off the years, line 217 on page 9.

**Editor:**

1. **Comment:**  Please ensure that your manuscript meets PLOS ONE's style requirements, including those for file naming. The PLOS ONE style templates can be found at

<https://journals.plos.org/plosone/s/file?id=wjVg/PLOSOne_formatting_sample_main_body>.pdf and 
<https://journals.plos.org/plosone/s/file?id=ba62/PLOSOne_formatting_sample_title_authors_affiliations.pdf>

**Response:**  Thank you for your comment. We have worked through the manuscript to ensure that the manuscript meets PLOS ONE’s style requirements.

2. **Comment:**  As required by our policy on Data Availability, please ensure your manuscript or supplementary information includes the following:
   - A numbered table of all studies identified in the literature search, including those that were excluded from the analyses.
     - For every excluded study, the table should list the reason(s) for exclusion.
     - If any of the included studies are unpublished, include a link (URL) to the primary source or detailed information about how the content can be accessed.
   - A table of all data extracted from the primary research sources for the systematic review and/or meta-analysis. The table must include the following information for each study:
     - Name of data extractors and date of data extraction .
     - Confirmation that the study was eligible to be included in the review.
     - All data extracted from each study for the reported systematic review and/or meta-analysis that would be needed to replicate your analyses.
     - If data or supporting information were obtained from another source (e.g. correspondence with the author of the original research article), please provide the source of data and dates on which the data/information were obtained by your research group.
     - If applicable for your analysis, a table showing the completed risk of bias and quality/certainty assessments for each study or outcome.  Please ensure this is provided for each domain or parameter assessed. For example, if you used the Cochrane risk-of-bias tool for randomized trials, provide answers to each of the signalling questions for each study. If you used GRADE to assess certainty of evidence, provide judgements about each of the quality of evidence factor. This should be provided for each outcome.
     - An explanation of how missing data were handled.

**Response:**  Thank you for pointing this out. We are pleased to provide the following two tables in the supplementary material:

- A numbered table of all studies found in the literature search including excluded studies with the reason for exclusion (S4 table).
- A table with all extracted data, including the required information on the date of extraction, the person who performed the extraction and confirmation that the study was eligible to be included (S6 Table).
  - There was no supporting information obtained by the authors from other sources.
  - Regarding the risk of bias and quality/certainty assessments for each study, all information is provided in Table 2, page 15f, and in the supplementary information (S5 Table).
  - We also included information on how the various authors handled missing data in their research to the S6 table.

3. **Comment:** Thank you for stating the following financial disclosure:

"This project is funded by the Gesellschaft für Forschungsförderung Niederösterreich m.b.H. (GFF) as part of the RTI-Strategy 2027 (Grant: FTI21-P-005). The funder's website: https://www.gff-noe.at/. GFF had no influence on the research or publication process.

This review is part of the CROB project (Collaborative Research on Occupational Balance), which is a research collaboration between the IMC University of Applied Sciences Krems (Austria), Duervation (Austria) and Karolinska Institutet (Sweden)."

Please state what role the funders took in the study.  If the funders had no role, please state: ""The funders had no role in study design, data collection and analysis, decision to publish, or preparation of the manuscript.""

If this statement is not correct you must amend it as needed.

Please include this amended Role of Funder statement in your cover letter; we will change the online submission form on your behalf.

**Response:** Thank you for this comment. We have added the disclosure statement to our cover letter.

4. **Comment:** Please include captions for your Supporting Information files at the end of your manuscript, and update any in-text citations to match accordingly. Please see our Supporting Information guidelines for more information: http://journals.plos.org/plosone/s/supporting-information.
   **Response:** Thank you for raising awareness for this. We have inserted captions for the Supporting Information.

We hope that our revisions meet your expectations and address your concerns. Thank you once again for your constructive feedback.

**Best regards,**

**Stefanie Lentner**
