# Supplementary material for: Interventions promoting occupational balance in adults: A systematic literature review
Source: PLoS One. 2025 Jun 5;20(6):e0325061. doi: 10.1371/journal.pone.0325061 (PMC12176295; doi:10.1371/journal.pone.0325061)
Supplement: Attachment — Submitted filename: Resonse to Reviewers.docx [file pone.0325061.s010.docx]

**Dear Reviewers and Dear Editor,**

Thank you for your valuable feedback on our manuscript. We appreciate the time and effort you have taken to review our work. We have thoroughly addressed all your comments and believe that your input has again significantly improved the manuscript. Below, we have responded to each of your comments in detail.

1. **Comment:** A small thing to start with: You use the word ‘compromises’ in two different places in the manuscript. I think you mean ’comprises’. (But on page 26, line 440, ‘compromising’ is used correctly.)

**Response:** Thank you for making us aware of the incorrect use of the word. We have corrected it accordingly.

2. **Comment:** These sentences, page 8, lines 188-191, make me confused: “The presentation of the results compromises an overview of the study characteristics, and the quality assessed, a brief description of the interventions and their effectiveness. In line with the two research questions the results are structured into the presentation of interventions addressing occupational balance, and an appraisal of their effectiveness.” It seems to me that you present two ways of organizing the results. Besides, the first sentence is not grammatically correct. Based on how your results are actually presented, I suggest the following: “The presentation of the results comprises an overview of the study characteristics, a quality assessment, and a brief description of the interventions and their effectiveness. In line with the two research questions, the results are structured into the presentation of interventions addressing occupational balance, and an appraisal of their effectiveness.”

**Response:** We appreciate your feedback and have revised the introductory sentence accordingly, page 8, line 190-193: “The results section provides an overview of the study characteristics and a quality assessment of included studies. In line with the two research questions, it further comprises a presentation of interventions addressing occupational balance, and an appraisal of their effectiveness.”

3. **Comment:** There is misuse of words like ‘hence’ and thus’. Delete as many as possible, but especially ‘thus’ on page 9, line 205, and substitute it with ‘and’. You already have ‘hence’ in the same sentence.

**Response:** Thank you for pointing out the misuse of these words. We have carefully reviewed each instance throughout the entire manuscript and made the necessary replacements in the following places: line 38: “therefore”, line 101: “to date”, line 110: “therefore”, table 3: “thereby”.

4. **Comment:** The number of participants in the BEL RCT is still not correct. It says very clearly in the paper from 2017 that there were 223 participants. I also think it is incorrect to view it as three RCTs if the papers are based on the same sample. It is quite common to write more than one paper from an RCT.

**Response:** Thank you for your comment, which suggests that we have not yet described this clearly. As different authors report sample sizes at different time points in the context of *BEL* (and other included studies), we have tried to be as consistent as possible in this review. Accordingly, we initially decided to report the sample size of all studies based on the endpoint (i.e., the number of participants actually analyzed). While we had stated this in Table 1 (notes) in the previous version of the manuscript, your feedback highlights the need to be more explicit about reporting sample sizes in this review. We have now decided to report both the initial sample size at baseline (*N*) and the final number of cases analyzed (*n* analyzed) at the endpoint of studies. We have now clarified the rationale for this approach in several sections of the manuscript:

- In the method section, we describe the reasoning for presenting both sample sizes, lines 178-181: “Table 1 presents both the sample size at baseline (*N*) and the final sample size (*n* analyzed), which is the number of participants included in the analyses after accounting for dropouts. This ensures that the review is based on the actual number of participants who completed all relevant measurements (53).”
- Again, we report both sample sizes when we mention the number of participants in the 'Study characteristics' section, lines 215-218: “The sample size at baseline (*N*) varied between 12 (67) and 226 (37) participants across all studies, involving diverse populations, which are described in detail below. A total of 641 study participants completed all study measurements and were included in the analyses at the study endpoints (i.e., *n* analyzed).”
- In the description of sample sizes in Table 1 we now report both the initial sample size at baseline (*N*) and the final sample size in terms of the total number of participants analyzed (*n* analyzed), see column 5 of table 1, pp. 11-14.
- We have subsequently removed the number of trial participants from the abstract (line 16) so as not to mislead readers without additional information.
- When describing research on *BEL* and *TTM* starting from line 289ff, we are more specific about the sample sizes. *BEL*: In line 290 we have indicated the sample size at the start of the study. *TTM*: we state the sample size at baseline in line 304.

We trust that our approach is now presented in a clear and comprehensible manner.

In response to your valid observation that it is methodologically inaccurate to treat the studies as three distinct RCTs when they are based on the same sample, we have implemented the following modifications to enhance clarity.

- To provide further precision, we added the phrase 'of the included publications' (line 213) in the list of study designs, ensuring that the total number provided aligns with the number of included publications.
- For references that utilize data from the same study population as other cited references (for both *BEL* and *TTM*), Table 1 (pp. 11-14) includes notes indicating, “Analyses from the same study cohort as in (37) OR (58)” or “The study cohort is part of the cohort in (37)”
- Under the heading “the effectiveness of interventions targeting occupational balance” (starting on page 20, line 287), we have provided a more detailed explanation indicating that these are distinct publications derived from a single RCT study (for both *BEL* and *TTM*).

5. **Comment:** On page 21, lines 303 – 307, you have this long sentence: “The results indicated that both the experimental and control group, which showed no significant difference between the groups at baseline, improved on all outcomes measured from pre- to post-intervention and were therefore on par, with improvements in occupational balance scores (COPM and OBQ) being statistically significant (p ≤ 0.01) in both groups (57).” I am not sure I understand what you mean. I suggest you split the sentence into two and insert a dot after ‘intervention’. Then please rewrite the last part of the sentence for clarity.

**Response:** Thank you for highlighting this issue. In response to your suggestion, we have revised the sentence by splitting it and rephrasing the latter part for enhanced clarity, as follows: (lines 307-311): “The results of the original RCT conducted in 2018 indicated that both the experimental and control group improved on all outcomes measured from pre- to post-intervention. The improvements in occupational balance scores (COPM and OBQ) were statistically significant (p ≤ 0.01) in both groups, indicating that the intervention was not significantly better than regular occupational therapy (58).”

6. **Comment:** The section starting on page 23, line 355, made me ponder a bit. I get the impression that social relations and digital solutions are something the authors are passionate about. It makes me think of argumentation rather than discussion of research findings. Okay, you give a reference to why social relations are important to occupational engagement (ref #60). But you gave no underpinnings to why a digital solution would be particularly suitable for an occupational balance intervention. Just your own thoughts and reasoning, which may be found true if tested, or perhaps not? I think you should nuance your text here. And perhaps move it down a bit and start with discussing what knowledge the articles have actually provided.

**Response:** Thank you for this valuable comment. We appreciate your insight and have revised the discussion section accordingly. We have made efforts to formulate the thematic emphasis on digital solutions and social relations (see lines 369 - 374, lines 410 - 412). To soften the emphasis on social relations, we have moved this section further back and rewritten it to present a more nuanced perspective.

7. **Comment:** Page 24, line 399, you have the year (2018) instead of a number for the reference.

**Response:** Thank you for pointing this out. We have now correctly cited the sentence (line 388). Additionally, we have carefully reviewed the entire manuscript and corrected similar instances in lines 61, 66, 205, 294, 298, and 312.

8. **Comment:** Please rewrite the sentence on page 26, lines 433-436, for clarity. Possibly a ‘which’ is missing on line 434.

**Response:** Thank you for this comment. We have reformulated the sentence for clarity (see lines 426 - 429): “As another example, two studies that reported beneficial effects for the AOI intervention and the ballroom dancing intervention, were rated as low quality due to small sample sizes, significant dropout rates and the use of a non-validated outcome measure.”

9. **Comment:** Page 26, line 451: You write ‘arguable’. Do you mean ‘inarguable’?

**Response:** Thank you for this comment. We implemented your suggestion (line 443).

10. **Comment:** Page 28, lines 487-491: Are you criticizing the studies you have reviewed for not being community-based, low-threshold services, or digital solutions? Or do you mean that existing research/interventions should be supplemented with such interventions? (I think many of the included interventions are in fact community based.) Again, I get the impression that you are more interested in conveying some messages than in discussing your findings. I think you could balance that a bit better. I absolutely agree with your arguments, as a citizen and human being; it is the academic researcher in me that protests.

**Response:** We fully concur with your observation. Thank you for highlighting this important issue. We have revised the section, accordingly, lines 479 - 486: “Scholars agree that interventions addressing occupational balance may be promising from a public health perspective (13, 34). While some interventions followed a community-based approach, most of them have been explored in clinical in- or outpatient healthcare settings or were delivered by institutions outside the healthcare sector, e.g., universities. Future research should supplement existing interventions with more community-based, low-threshold services, or digital solutions. Expanding the scope to include more diverse and accessible formats will help in increasing their public health impact.”

11. **Comment:** Page 29, lines 512-513, you write that qualitative and mixed-methods research was not included in the literature review. Participatory research may fall under those categories. It seems a bit illogical to argue, as you indicate on page 28, lines 502-503, that participatory research is missing, since the reason may be that you have excluded that type of research. It is perhaps not likely, but you cannot know.

**Response:** We appreciate your observation, thank you. To clarify and avoid any perceived contradiction, we have reorganized the argumentation. We acknowledge that excluding participatory research is a limitation of our review. Consequently, we have linked these points and grouped them under the heading of limitations, explicitly stating that the absence of qualitative research in our review is considered a limitation, lines 501 - 515: “Due to the chosen methodology, interventions studied using qualitative or mixed-method designs were not included in this review. Acknowledging the subjectiveness of the occupational balance concept as well as the fact that it may differ across cultures, age groups and populations is crucial. Future projects aiming to design occupational balance interventions may follow participatory research approaches and truly involve the population of interest throughout the design process. In fact, additional interventions promoting occupational balance were detected, which still need to be examined for their effectiveness with quantitative methods. Among the interventions discovered were Project Bien Estar (100), self-management occupational therapy program (SMOoTh) (101), educational workshop on time use (102), inpatient energy management education (IEME) (103), mindful based program (104), home modification intervention (105), psychological rehabilitation program (106), therapeutic gardening (33) and Daily Life Coping (107). Given that these interventions demonstrate promising approaches and existing research may already incorporate the aforementioned participatory methods, further investigation is warranted.”

12. **Comments to the authors:** The manuscript ist not written in standard English / language issues

**Response**: The entire CROB research team and a native speaker have reviewed the manuscript linguistically and made improvements at several points, all of which have been marked in the manuscript for tracking.

13. **Journal requirements:** Please review your reference list to ensure that it is complete and correct. If you have cited papers that have been retracted, please include the rationale for doing so in the manuscript text, or remove these references and replace them with relevant current references. Any changes to the reference list should be mentioned in the rebuttal letter that accompanies your revised manuscript. If you need to cite a retracted article, indicate the article’s retracted status in the References list and also include a citation and full reference for the retraction notice.

**Response**: We have checked the reference list for retracted entries, and no references needed to be corrected.

We hope that our revisions align with your expectations and effectively address your concerns. Thank you once again for your insightful feedback.

**Best regards,**

Stefanie Lentner
